# Self-assembled π-conjugated Cu(ii)–phenanthro[9,10-*d*]imidazole superstructures for VOC sensing and enhanced supercapacitor performance

**DOI:** 10.1039/d5na00758e

**Published:** 2025-09-23

**Authors:** Mallayasamy Siva, Aneesh Anand Nechikott, Sheethal Sasi, Yuvaraj Sivalingam, Prasant Kumar Nayak, Priyadip Das

**Affiliations:** a Department of Chemistry, SRM Institute of Science and Technology SRM Nagar, Potheri, Kattankulathur 603203 Tamil Nadu India priyadipcsmcri@gmail.com priyadip@srmist.edu.in prasantnayak15@gmail.com prasantn1@srmist.edu.in; b Department of Physics and Nanotechnology, Faculty of Engineering and Technology, SRM Institute of Science and Technology Kattankulathur 603203 Tamil Nadu India; c Centre for Advanced Translational Research, KPR College of Arts Science and Research Avinashi Road, Arasur Coimbatore 641407 Tamil Nadu India yuvaraj.sst@gmail.com yuvaraj.s@kprcas.ac.in

## Abstract

The development of self-assembled smart materials is a pivotal area of advanced research, particularly for sensing and electronic applications. π-Conjugated small organic molecules can self-assemble into well-ordered superstructures with remarkable optoelectronic, chemical, and structural properties, making them suitable for applications such as volatile organic compound (VOC) detection and energy storage in supercapacitors. However, the self-assembly behavior of Cu(ii) complexes derived from π-conjugated ligands, and their potential use in areas such as health, environmental monitoring, and energy storage, remain underexplored. In this study, we designed and synthesized two π-conjugated phenanthro[9,10-*d*]imidazole-based ligands (S1 and S2) and their corresponding Cu(ii) complexes, (S1)_2_Cu and (S2)_2_Cu. These complexes self-assemble into well-ordered superstructures with distinct morphologies and selectively detect acetone vapors *via* Scanning Kelvin Probe (SKP) measurements. Their properties are governed by multiple non-covalent interactions in combination with metal–ligand coordination, which control the shape and size of the assemblies. Surface photovoltage measurements under dark and UV conditions, in the presence of different VOC vapors, revealed that (S1)_2_Cu exhibits superior selectivity toward acetone compared to (S2)_2_Cu. The pseudo-capacitive performance of the self-assembled superstructures was also evaluated in 1.0 M KOH aqueous electrolyte, yielding specific capacitances of 230.0 F g^−1^ for (S1)_2_Cu and 195.0 F g^−1^ for (S2)_2_Cu. (S1)_2_Cu also demonstrated higher rate capability and better capacitance retention (75% after 4000 cycles). Overall, this work presents a promising strategy for designing self-assembled superstructures from metal-coordinated π-conjugated systems as advanced functional materials for VOC sensing and potential electrode materials for aqueous supercapacitor applications.

## Abstract 2

Cu(ii) complexes of S1 and S2, self-assembled into well-ordered superstructures present promising smart materials for acetone sensing, offering potential applications in designing VOC sensor devices and supercapacitor applications.

## Graphical abstract

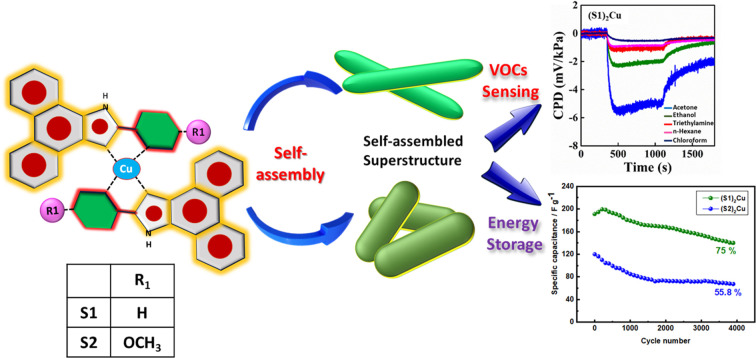

## Introduction

Self-assembly of π-conjugated systems offers a promising route to materials with properties markedly different from those of their monomeric forms, as their functional characteristics are governed by electronic coupling between molecular building blocks.^1–3^ Well-ordered structures from π-conjugated molecules have attracted significant attention due to their unique optical and electronic properties,^4–6^ making them excellent candidates for molecular electronics. Small π-conjugated molecules with suitable chromogenic units can form highly ordered superstructures with distinctive optical and electronic characteristics,^7,8^ but integrating these architectures into devices remains challenging due to the need for controlled interchain electronic coupling.^9^

Understanding the self-assembly behaviour and fabrication-induced optical characteristics of π-conjugated systems is essential.^10–13^ Functionalized π-conjugated small molecules are of great interest in nanoscience^14–21^ owing to their synthetic flexibility, diverse geometries, and tuneable structures.^22–26^ While conventional self-assembly of small π-conjugated molecules has been widely studied, metal-coordinated π-conjugated systems are still emerging. The incorporation of metal ions introduces new structural topologies and interaction sites, offering enhanced control over morphology, stability, and device performance.^27,28^ In particular, metal-coordinated small aromatic π-conjugated molecules can self-assemble into well-ordered structures with distinctive properties, making them promising for applications in molecular electronics, catalysis, energy conversion, and sensing.^29–34^ In our recent work, we synthesized six phenanthro[9,10-*d*]imidazole-based Zn(ii) and Cd(ii) complexes and explored their self-assembly.^35^ These structures exhibit exceptional VOC sensing capabilities, effectively detecting acetone, formaldehyde, benzene, and toluene in various environments. Notably, the Cd(ii) complexes selectively detect acetone – an important biomarker for diabetes and an industrial solvent-underscoring their potential for health diagnostics and environmental monitoring. Copper-coordinated π-conjugated systems spontaneously self-assemble through interactions between metal ions and ligands, forming complex structures. These systems often display unique properties such as aggregation-induced emission and stimuli responsiveness.^36–38^ Coordination with Cu(ii), a d^9^ system, notably affects the optical and electrical properties of the assembled state, shaping their potential applications in specialized fields. Yan-Hu designed and synthesized a novel anthracene-based ligand, which upon Cu(ii) mediated self-assembly generate luminescent supramolecular coordination compounds.^39^ In this regard, Tandon *et al.* described the self-assembly of antiferromagnetically coupled Cu(ii) supramolecular architectures with diverse structural complexities.^40^ Recently, Tong *et al.* reported the design of an eight-coordinate (8C) Cu(ii) heterometallic complex, displaying a distorted dodecahedral structure with an [(O_2_)_4_] donor set, which has been synthesized by programmable self-assembly.^41^ However, the self-assembly properties and mechanisms of Cu(ii)-coordinated small π-conjugated organic molecules, as well as their potential applications, remain underexplored. Therefore, we aim to investigate the self-assembly behavior of Cu(ii)-coordinated phenanthro[9,10-*d*]imidazole-based π-conjugated ligands previously synthesized and explore their potential in emerging research fields.

**Fig. 1:**
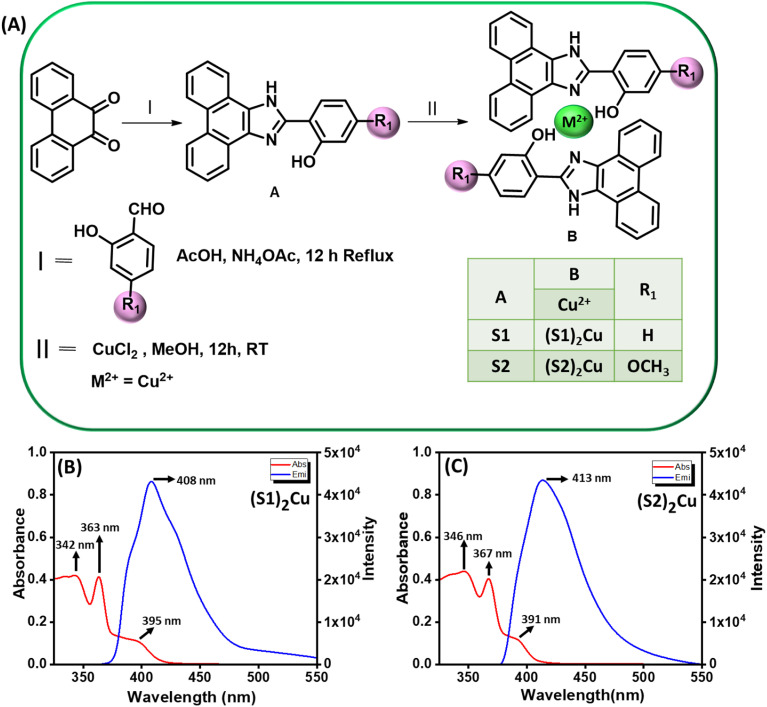
(A) Methodology adopted for the synthesis of S1, S2 and their corresponding metal complexes (S1)_2_M and (S2)_2_M where M = Cu(ii). Absorption and steady-state emission spectra of (B) (S1)_2_Cu, and (C) (S2)_2_Cu in a 50% aqueous-ACN medium.

We report the synthesis of two Cu(ii) complexes, (S1)_2_Cu and (S2)_2_Cu, derived from phenanthro[9,10-*d*]imidazole-based ligands S1 and S2 (Fig. 1A). These complexes exhibit self-assembly behavior, forming diverse superstructures with distinct morphologies. To evaluate their surface potential and gas adsorption, surface photovoltage measurements were conducted using an SKP setup. We also examined their photo-induced charge transport and gas adsorption properties under both light and dark conditions, testing various VOCs. The results revealed that the self-assembled Cu(ii) complexes show notable sensitivity and selectivity for acetone. Supercapacitors bridge the gap between high-energy density batteries and high-power density conventional capacitors, complementing battery technology.^42–46^ They are classified as electrical double-layer capacitors (EDLCs) or pseudocapacitors based on their charge storage mechanisms. Carbon-based materials exhibit EDLC behavior with capacitances of 100–200 F g^−1^,^47^ while transition metal oxides and conducting polymers show pseudocapacitance above 200 F g^−1^.^48^ Although RuO_2_ offers high capacitance (∼700–800 F g^−1^), its cost, scarcity, and toxicity limit its use, prompting exploration of alternatives like MnO_2_, NiO, CuO, and V_2_O_5_.^49–53^ Copper-based oxides and hydroxides are particularly attractive due to their abundance, low toxicity, and environmental friendliness^52,54,55^ with reported capacitances ranging from 200 to 500 F g^−1^ in alkaline electrolytes.^56–59^ However, the use of Cu-complexes derived from π-conjugated organic molecules in supercapacitors remains underexplored. Here, we investigate the electrochemical capacitance performance of self-assembled Cu(ii) complexes (S1)_2_Cu and (S2)_2_Cu in 1.0 M KOH, measuring specific capacitances *via* galvanostatic charge–discharge (GCD) cycling in the voltage range of 0–0.6 V at a specific current of 1 A g^−1^. Interestingly, (S1)_2_Cu and (S2)_2_Cu delivered specific capacitances of 230.0 F g^−1^ and 195.0 F g^−1^, respectively. Moreover, (S1)_2_Cu exhibited a superior capacitance retention of 42.0% when cycled at a higher specific current of 20 A g^−1^ by delivering a specific capacitance of about 96.6 F g^−1^, whereas the capacitance retention of (S2)_2_Cu is only 37.9% with a specific capacitance of about 74.0 F g^−1^ at a specific current of 12 A g^−1^. This result indicates the superior rate performance of (S1)_2_Cu compared to that of (S2)_2_Cu for supercapacitor application.

## Results and discussion

We designed and synthesized two Cu(ii) complexes ((S1)_2_M, and (S2)_2_M where M = Cu(ii)) from previously synthesized ligands S1 and S2.^60^ The corresponding Cu(ii) complexes were synthesized by the reaction of an aqueous solution of CuCl_2_ with methanolic solutions of S1 and S2 (Fig. 1A). These copper(ii) complexes are isolated as pure solids. To confirm their structural integrity and purity, both ligands and metal complexes undergo several characterisation studies using various standard analytical and spectroscopic techniques (SI Fig. S1–S8). We recorded the UV-vis absorption and steady-state emission spectra of copper(ii) complexes ((S1)_2_Cu and (S2)_2_Cu) at room temperature in a 50% aqueous-ACN medium. The UV-vis absorption spectra of (S1)_2_Cu (terminal 2-hydroxy phenyl group) exhibited an absorption maximum at 395 nm (*ε* = 5.6 × 10^3^ M^−1^ cm^−1^) along with other shorter peaks at 363 and 342 nm (Fig. 1B). The absorption maximum at 395 nm is possibly due to the metal-to-ligand charge transfer (MLCT) transition facilitated by the π-acceptance property of the phenanthro[9,10-*d*]imidazole system.^61,62^ The other shoulder peak at 363 nm may be ascribed to the spin-allowed intraligand charge transfer (ICT) process 
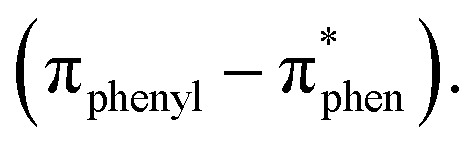
^63,64^ The other shorter wavelength absorption band at 342 nm is probably due to the other π–π* transitions associated with the π-conjugated molecular backbone.^65^ Similarly, (S2)_2_Cu (terminal 2-hydroxy 4-methoxy phenyl group) exhibited the metal-to-ligand (MLCT) based absorption maximum at 391 nm (*ε* = 6.3 × 10^3^ M^−1^ cm^−1^) with additional absorption peaks at 367 and 346 nm associated with ICT and other π–π* based transitions (Fig. 1C).

It is already recognized that introduction of electron-donating groups (EDGs), such as –OCH_3_ can significantly decrease the electron affinity of the ligand (S2). Therefore, substitution of the phenyl ring at the 2-position of the imidazole ring in the phenanthro [9,10-*d*] with EDGs can influence the electron-accepting properties of the ligand, which effectually alter the energy gap associated with metal-to-ligand charge transfer (MLCT) and intramolecular charge transfer (CT) transitions. As a result, we observed a noticeable blue shift of the characteristic MLCT band of (S2)_2_Cu (391 nm) compared to (S1)_2_Cu (395 nm). We also recorded the steady state luminescence spectra of these copper(ii) complexes: (S1)_2_Cu and (S2)_2_Cu. The steady-state emission spectra of (S1)_2_Cu and(S2)_2_Cu exhibited the metal to ligand charge transfer emission peaks at 408 nm (*λ*_EX_ = 363 nm) and 413 nm (*λ*_EX_ = 367 nm), respectively (Fig. 1B and C). The calculated quantum yields of these Cu(ii) complexes at their respective ICT based emission maxima are tabulated in Table 1. The emission spectral analysis suggests that the presence of an electron-donating group (EDG) in the π-conjugated backbone of the ligand is responsible for the effectual alteration of the frontier molecular orbitals (FMOs) associated with the MLCT emission band, which is responsible for the red shift of the characteristic MLCT based emission band of (S2)_2_Cu compared to (S1)2Cu without any EDG substitution.

**Table 1:** Quantum yield measurement of (S1)_2_Cu and (S2)_2_Cu in a 50% aqueous-ACN medium

Metal complexes	Excitation wavelength (*λ*_Ex_) (nm)	Monitoring wavelength (*λ*_Em_) (nm)	Quantum yield (*Φ*_F_) (%)
(S1)_2_Cu	363 nm	408 nm	7.85%
(S2)_2_Cu	367 nm	413 nm	16.92%

Then, we have studied the self-assembly properties of these Cu(ii) complexes: (S1)_2_Cu and (S2)_2_Cu in a 90% aqueous-ACN medium.

**Fig. 2:**
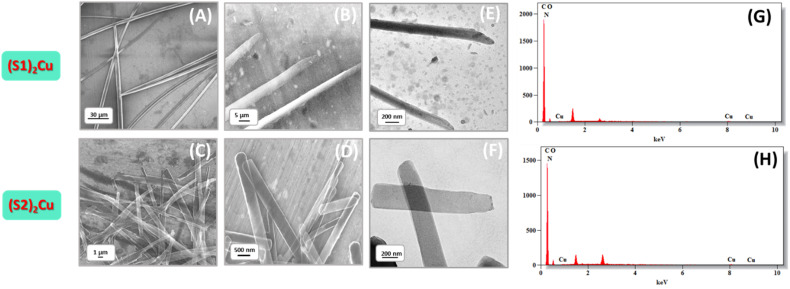
Microscopic analysis of the self-assembled structure of Cu(ii) complexes: (S1)_2_Cu and (S2)_2_Cu. HR-SEM images of the self-assembled structures formed by (S1)_2_Cu (A & B) and (S2)_2_Cu (C & D) in a 90% aqueous-ACN medium. TEM micrographs of the self-assembled superstructures of (S1)_2_Cu (E) and (S2)_2_Cu (F) in a 90% aqueous-ACN medium. EDX analysis of (G) (S1)_2_Cu and (H) (S2)_2_Cu (not assigned peak: Al and Cl).

The choice of a highly polar medium was due to its ability to facilitate the formation of self-assembled superstructures by through well-ordered assembly of monomeric building blocks. In order to trigger the self-assembly process of these Cu(ii) complexes (S1)_2_Cu and (S2)_2_Cu, we dissolved each complex in 1,1,1,3,3,3,3-hexafluoro-2-propanol (HFIP) to reach an initial concentration of 100 mg mL^−1^. Then, each solution was diluted with 90% aq-ACN solution to achieve a final concentration of 2 mg mL^−1^. High-resolution scanning electron microscopy (HR-SEM) revealed that (S1)_2_Cu self-assembles into needled shaped elongated nanorod like morphology (Fig. 2A and B). On the other hand, HR-SEM images showed that self-assembled (S2)_2_Cu displayed nanorod-like morphology (Fig. 2C and D). We have also performed high resolution transmission electron microscopy (HR-TEM) analysis of the self-assembled superstructures obtained from (S1)_2_Cu and (S2)_2_Cu, which is well in agreement with the HR-SEM analysis (Fig. 2E and F). The morphology of these self-assembled Cu(ii) complexes was quantitatively assessed by analysing the fibre and nanorod width and length distribution based on HR-SEM images. At lower magnifications, SEM images reveal that needled shaped elongated nanorods formed by (S1)_2_Cu are uniformly distributed and show a comparatively lower density. Needle-shaped elongated nanorods formed by (S1)_2_Cu have a similar length with an average value of approximately 340 ± 2 μm, while the nanorods derived from (S2)_2_Cu display shorter lengths having an average value of 180 ± 2 μm. Additionally, fibre width varies significantly depending on the ligand and metal compositions. In our case, the needle-shaped elongated nanorod obtained from (S1)_2_Cu has an average diameter of 329 ± 7 nm. On the other hand, the nanorods fabricated from (S2)_2_Cu exhibit a larger average diameter of 382 ± 4 nm. These variations in width and length reflect the influence of different metal–ligand combinations on the resulting nanostructure morphology (SI Fig. S9 & S10). We proposed that the finely balanced, geometrically constrained orientation arises from the metal-to-ligand interactions combined with the restricted molecular flexibility of the building blocks, and facilitate the π–π stacking interactions, which drive the overall self-assembly process. The morphological alteration among the superstructures obtained from different ligands with the same metal ions is due to differences in the electronic properties of the ligand with or without electron donating substitution at the 2 positions of the terminal phenol moiety of the phenanthro[9,10-*d*]imidazole scaffold. This variation impacts the electronic properties of the overall π-conjugated system and tunes the self-assembly process through π–π interactions. The presence of Cu(ii) in the self-assembled structures obtained from these Cu(ii) complexes was further verified through energy-dispersive X-ray spectroscopy (EDX) analysis (Fig. 2G and H). The self-assembly of (S1)_2_Cu and (S2)_2_Cu is driven by a combination of metal–ligand coordination, π–π interactions, hydrogen bonding, and solvophobic effects, which together direct the hierarchical organization of the molecules. Cu(ii) coordination with the imidazole-phenanthrene ligands induces planarity in the conjugated backbone, promoting π–π stacking and the formation of lamellar molecular arrangements. Lateral association *via* C–H⋯O hydrogen bonding and van der Waals forces further support the lateral association of these molecular sheets, resulting in intermediate lamellar assemblies, as evidenced by PXRD data.

To minimize interfacial free energy, these lamellar sheets subsequently undergo a scrolling or rolling process, giving rise to elongated nanorod structures. The differences in substituents – hydroxyl (–OH) in (S1)_2_Cu and both hydroxyl (–OH) and methoxy (–OCH_3_) in (S2)_2_Cu – influence packing density and introduce varying degrees of torsional strain. These structural variations account for the distinct morphological features observed in the electron microscopy images.

**Fig. 3:**
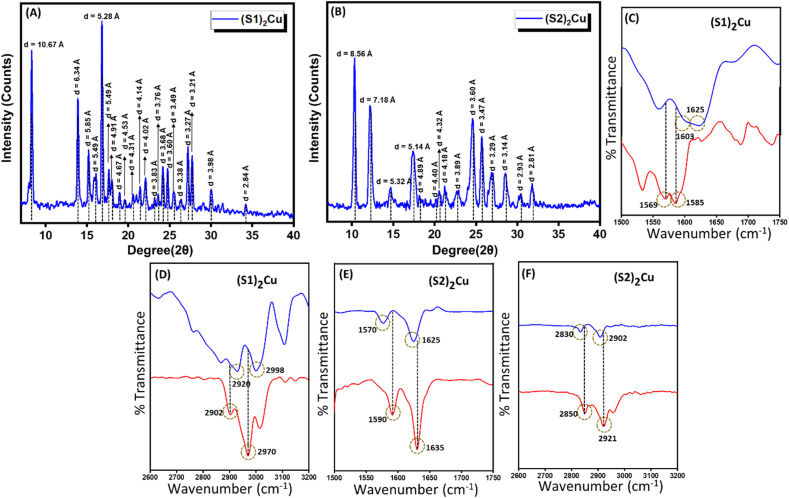
PXRD of the self-assembled structure formed by (A) (S1)_2_Cu and (B) (S2)_2_Cu in a 90% aqueous-ACN medium. Concentration-dependent FT-IR spectra of (C) & (D) (S1)_2_Cu and (E) & (F) (S2)_2_Cu, as concentration varies (red – 0.5 mg mL^−1^); (blue – 2.5 mg mL^−1^).

**Scheme 1:**
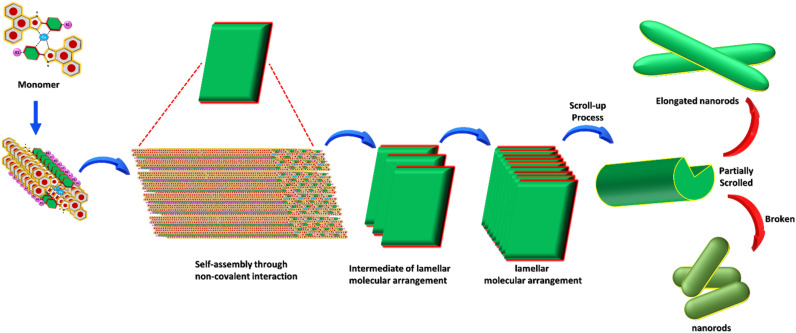
A schematic illustration of the formation of elongated nanorods and nanorods by the self-assembly of (S1)_2_Cu and (S2)_2_Cu in a 90% aqueous-ACN medium through an intermediate lamellar molecular arrangement followed by layer closure or the scroll-up process.

The aggregation behaviour of these two Cu(ii) complexes (S1)_2_Cu and (S2)_2_Cu in their self-assembled state was further investigated through Powder X-ray Diffraction (PXRD) analysis (Fig. 3A and B) of the dried mass of these Cu(ii) complexes, prepared from a 90% aqueous-ACN solvent mixture. The PXRD patterns of (S1)_2_M and (S2)_2_M (M = Cu(ii)) showed prominent sharp peaks characteristic of well-ordered crystalline structures within a wide 2*θ* range between 3° and 50°. Quantitatively, the degree of crystallinity of (S2)_2_Cu was estimated to be 78.86%, which is significantly lower than the degree of crystallinity calculated for (S1)_2_Cu (56.55%) (SI Table S1). In the wide-angle region, the PXRD spectrum of (S1)_2_Cu displayed two distinct peaks at 19.5° and 20.1° (*d*-spacing values of 4.89 Å and 4.40 Å, respectively) (Fig. 3A). Similarly, peaks at 20.1° and 20.5° (with *d*-spacing values of 4.41 Å and 4.32 Å, respectively) were also identified for (S2)_2_Cu (Fig. 3B). Presence of these peaks clearly suggests the presence of π–π stacking interactions between the π-conjugated molecular building blocks. Characteristic peaks at 24.1° and 27.7° (*d*-spacing values of 3.68 Å and 3.21 Å) for (S1)_2_Cu, and at 24.7° and 27.0° (*d*-spacing values of 3.60 Å and 3.29 Å) for (S2)_2_Cu (Fig. 3A) evidently suggest the existence of intermolecular H-bonding involving imidazole NH with the solvent molecules. Moreover, the PXRD spectrum of the nanorod structures formed by (S2)_2_Cu displayed a number of diffraction peaks at 5.97°, 12.31°, 18.13°, 22.80°, and 30.45 with corresponding spacing values of 7.18 Å, 4.89 Å, 3.89 Å, 3.60 Å, and 2.93 Å, respectively. The observed periodic ratios of 1/2, 1/3, 1/4, and 1/5 strongly suggest the formation of a lamellar molecular arrangement during the self-assembly process of (S2)_2_Cu (Fig. 3B). This ordered lamellar arrangement is driven by the interplay of noncovalent interactions, mainly π–π stacking and intermolecular hydrogen bonding, ensuring the stability of the molecular assembly. In a highly polar aqueous medium, the lamellar molecular arrangements underwent a scroll-up process to get an energetically stable structure. This scroll-up process not only protects the π-conjugated molecular backbone from hydrophobic interactions but also minimizes the potential surface energy of the self-assembled superstructures, which sometimes triggered the breakage of self-assembled nanorods into small pieces.^66^ This transformation highlights the significant influence of the solvent environment and noncovalent interactions in governing the morphology and stability of the self-assembled structures (Scheme 1).

To gain a deeper understanding about the role of non-covalent interactions, specifically intermolecular hydrogen bonding and π–π stacking, in the self-assembled state, concentration-dependent FT-IR analysis was performed for the dried mass of these two Cu(ii) complexes of S1 and S2 in their self-assembled states obtained from a 90% aqueous-ACN mixture with two distinct concentrations of 0.5 mg mL^−1^ and 2.5 mg mL^−1^ respectively. The FT-IR spectra of these Cu(ii) complexes displayed several characteristic peaks, which were ascribed to functional groups, like C=C (aromatic peak value), C=N (aromatic peak value), C–H (aromatic, peak value), and imidazole N–H vibrations (peak value). As the concentration of these metal complexes increased, significant changes in the peak position and peak intensity of these characteristic peaks were observed (Fig. 3C–F). (SI Table S2). This alteration in the peak position and peak intensity evidently confirmed the presence of intermolecular H-bonding and π–π stacking in the self-assembled state of these Cu(ii) complexes. The above experimental results evidently proposed that subtle balance of non-covalent interactions mainly π–π stacking and intermolecular H-bonding along with the molecular flexibility governed the preferred conformations of these Cu(ii) complexes for self-assembly to generate well-ordered superstructures. In order to check the thermal stability, we have also performed the thermogravimetric analysis (TGA) of the self-assembled superstructures obtained from (S1)_2_Cu and (S2)_2_Cu. The TGA showed that these (S1)_2_Cu and (S2)_2_Cu based superstructures are thermally stable up to ∼220 °C, ensuring the high thermal stability of these self-assembled structures (SI Fig. S11).

Furthermore, we checked the optoelectronic properties of these Cu(ii) complex based self-assembled superstructures by measuring the contact potential difference (CPD) using SKP both in the dark and in the presence of UV-light. The light-assisted surface voltage variations with VOC adsorption of the materials were studied with the help of a scanning Kelvin probe (SKP) system as reported in previous studies.^67–70^ The setup consists of a gold tip of 2 mm diameter that probes above the surface of the sample with a frequency of 78.3 Hz. An AC voltage Vac(*ω*) is applied to the gold tip and the contact potential difference (CPD) between the tip and the sample is measured and analysed. A standard gold sample (Au) was utilized in the system to calibrate the tip. To analyse the effect of gas adsorption on the surface of the samples, they were exposed to various volatile organic compounds (VOCs) at room temperature. The experiments were carried out as reported in previous literature studies.^67,71–74^ The sample preparation for SKP measurements is as follows. The fluorine-doped tin oxide (FTO) coated glass substrate was first cut into pieces (2 cm × 1 cm) and extensively cleaned with soap solution, distilled water, acetone, and ethanol in an ultra sonicator bath for 30 min at 55 °C. Chloroform was used to dissolve the samples before they were spin coated onto the prepared FTO substrate. The coated films were left to dry in air for 6 hours at room temperature (RT). Herein, the measurements were carried out for CPD between the sample and the conductive gold tip. All the experimental measurements were carried out in the dark and under UV illumination at RT (25 °C) in a closed chamber. The samples were illuminated with a UV light source of 365 nm wavelength. Various VOCs including ethanol, acetone, triethylamine (TEA), *n*-hexane and chloroform were employed to analyse the gas adsorption properties of the samples.

The CPD measurements with the SKP setup were not carried out by varying the concentration of each VOC. Instead, to maintain a comparison of the volatility, the obtained CPD values were normalized with the saturated vapour pressure (SVP) values of the corresponding VOCs at RT (25 °C). The work function (WF) changes of the samples were calculated after measuring the surface potential changes during gas adsorption using the equation relating CPD of the samples and that of gold.1
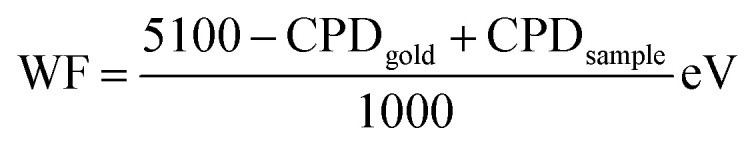
where CPD_gold_ and CPD_sample_ are the potential differences measured with respect to the gold tip for a reference gold sample and the examined material, respectively.

**Fig. 4:**
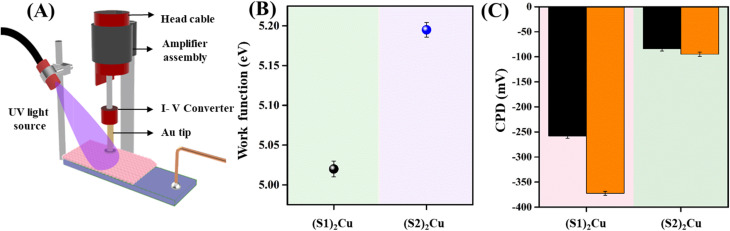
(A) Schematic illustration of the SKP setup, (B) work function measurements of the samples, and (C) CPD changes under dark (black) and UV light (orange) exposure for the samples in an ambient air medium.

The integer 5100 is the standard work function of gold in meV. Fig. 4A shows the schematic illustration of the SKP setup utilized to carry out gas adsorption studies. The CPD measurements of the samples in a dark medium in ambient air were performed from which the WF of the materials was calculated (Fig. 4B). The results show a comparatively decreased WF for (S1)_2_Cu samples than (S2)_2_Cu. A reduced WF helps in increased charge transfer during light and gas exposure. Furthermore, the measurements were performed under UV light exposure and the CPD changes of the samples in the dark and under UV light illumination in an ambient air medium are given in Fig. 4C. Even though both samples show an improvement in CPD upon light exposure in comparison to their dark counterparts, the highest CPD change arises for the (S1)_2_Cu sample. It can be seen that upon UV light illumination, (S1)_2_Cu showed a higher CPD change than (S2)_2_Cu. On exposure to UV light, photo-induced charge carriers are created improving the charge transfer process leading to an excess of electrons in the samples.

**Fig. 5:**
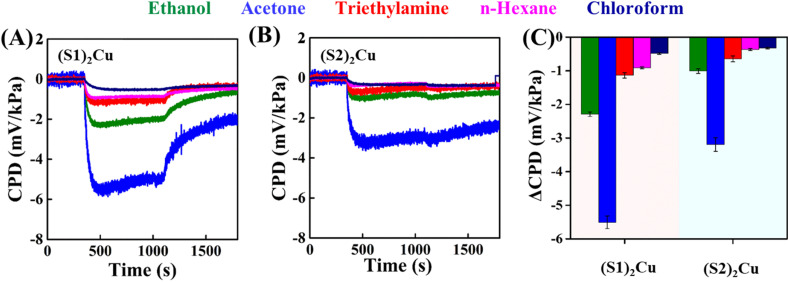
Time evolution of the changes in the CPD signal with UV light exposure under various VOC atmospheres for (A) (S1)_2_Cu and (B) (S2)_2_Cu and (C) delta CPD plots of (S1)_2_Cu and (S2)_2_Cu.

The coordination number is determined based on several factors, including (i) the size of the ligand, (ii) the size of the metal ion, (iii) the electronic configuration of the metal ion, and (iv) intermolecular interactions, which can vary between the solution and solid states. In our study, intermolecular interactions play a critical role in the acetone sensing ability of copper complexes. Specifically, the copper complex derived from phenanthrol-imidazole with 2-hydroxybenzene (S1)_2_Cu exhibits superior acetone sensing compared to that with 4-methoxy-2-hydroxybenzene (S2)_2_Cu. This enhanced acetone sensing ability is attributed to the stronger hydrogen bonding interactions facilitated by the NH group in imidazole and copper metal. These interactions create a more favourable environment for the adsorption and interaction of acetone molecules. In contrast, the presence of the methoxy group in the 4-methoxy-2-hydroxybenzene ligand introduces electron-donating effects, which reduce the hydrogen bonding capability of the phenolic moiety. Consequently, the copper complex with 2-hydroxybenzene is more effective in sensing acetone due to the increased strength of intermolecular interactions with the volatile organic compound. The attractive properties of the self-assembled superstructures make them promising towards gas adsorption studies on their surfaces. Consequently, the gas adsorption properties were analyzed using the SKP setup at RT under the influence of VOCs and UV light with an air medium kept as the reference. The SKP measurements are not concentration-based studies of VOCs. Instead, each VOC was allowed to vapourize for a fixed time, after which the measurements were performed. To maintain the volatility of the examined VOCs, the CPD in each case was normalized with the respective saturated vapour pressure (SVP) of the VOC. Antoine's eqn (2) was followed to calculate the SVP of the VOC at RT.^71^2
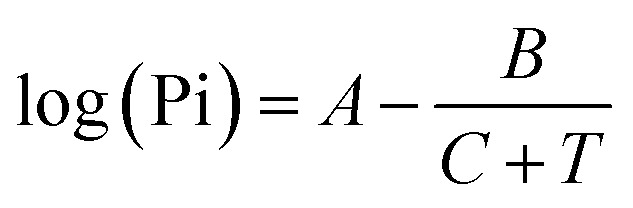
where Pi is the VOC's individual vapour pressure, *T* is temperature (here 25 °C) and *A*, *B* and *C* are the Antoine parameters. Our previous publications also show similar normalizations.^35,72^ The time evolution of CPD changes measured upon exposure of the samples to vapours of ethanol, acetone, TEA, *n*-hexane and chloroform with UV light OFF and ON is depicted in Fig. 5. On analyzing the results (Fig. 5A and B), it was observed that all the samples exhibit a n-type response towards VOCs on UV light exposure out of which the highest response was exhibited by (S1)_2_Cu samples selectively towards acetone in comparison to (S2)_2_Cu. (S2)_2_Cu shows a comparatively lower response than (S1)_2_Cu. Fig. 5C shows the changes in CPD of the samples in each VOC medium. All the samples are shown to exhibit a good response towards acetone vapours than to other VOCs which can be attributed to the electron cloud formation in acetone arising from their higher dipole moment. This helps in strong interaction and selective adsorption of the samples with acetone. We proposed that the superior and selective response of (S1)_2_Cu and (S2)_2_Cu toward acetone vapours can be attributed to the imidazole NH groups and Cu(ii) metal ions present on the surface of the self-assembled structures. These sites enable efficient interactions with acetone molecules through hydrogen bonding and coordination with the metal center.^35^ The response times of (S1)_2_Cu in acetone media were obtained to be 39 s and 50% recovery was obtained by this system in 185 s. The samples ((S1)_2_Cu and (S2)_2_Cu) show lesser response to ethanol, TEA, *n*-hexane and chloroform. This could be explained in terms of the lesser hydrogen bonding in ethanol leading to less adsorption.

The absence of hydrogen bonding in *n*-hexane and chloroform also makes them the least interacting. The bulky ethyl groups in TEA hinder its interaction with the sites of the samples, thus reducing its response. In effect, the VOCs ethanol, TEA, *n*-hexane and chloroform do not exhibit discriminable changes in CPD of the samples.

**Fig. 6:**
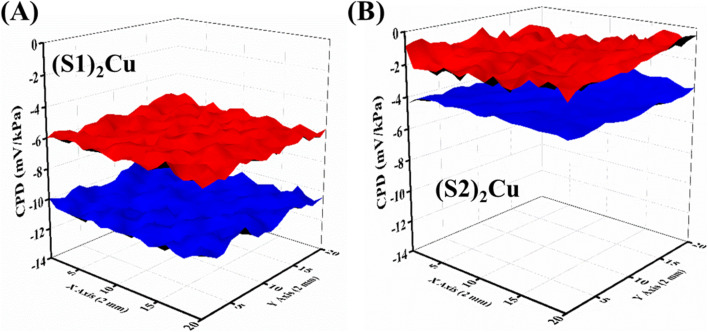
3D raster scan images under dark (red) and UV light (blue) conditions in an acetone medium for the samples (A) (S1)_2_Cu and (B) (S2)_2_Cu at room temperature.

The homogeneity of the samples and the effect of uniform VOC adsorption were confirmed by a 3D raster scan measurement of the sample surface (2 mm × 2 mm) in the SKP setup. The scan was performed in each VOC medium. The results in the acetone medium are depicted in Fig. 6 and those in the other VOCs are given in Fig. S12. The plots show the homogeneity of the sample surface upon acetone adsorption both in the dark and under UV light illumination. It also confirms that the photo-enhanced gas response in the acetone medium is higher for the (S1)_2_Cu sample compared to all other samples. Thus, a reasonably higher selectivity and sensitivity of the samples can be attributed to the (S1)_2_Cu sample than (S2)_2_Cu. Acetone is an important biomarker in the detection of diabetes mellitus, and can thus be detected using (S1)_2_Cu molecules. This paves the way for the fabrication of novel-gas sensors towards the detection of acetone in diagnosing diabetes mellitus by breath analysis in people.

### Electrochemical performance of (S1)_2_Cu and (S2)_2_Cu

**Fig. 7:**
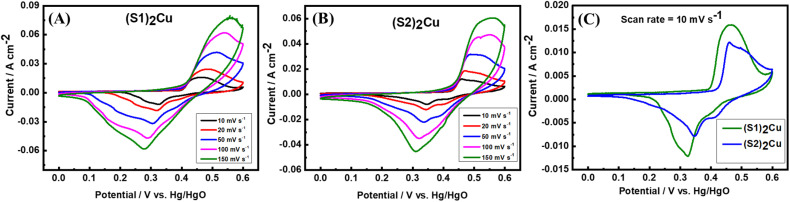
CVs of (A) (S1)_2_Cu and (B) (S2)_2_Cu at different scan rates varying from 10 mV s^−1^ to 150 mV s^−1^ in the aqueous electrolyte of 1.0 M KOH; (C) comparative CV of (S1)_2_Cu and (S2)_2_Cu at a scan rate of 10 mV s^−1^.

**Fig. 8:**
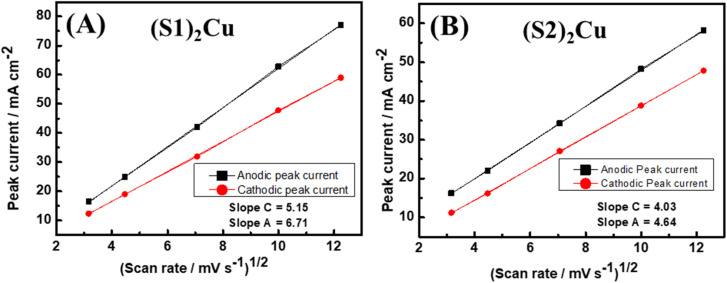
Plots of peak current *vs.* square root of scan rates for (A) (S1)_2_Cu and (B) (S2)_2_Cu measured at various scan rates.

Cyclic voltammetry (CV) is an essential electroanalytical technique to characterise the capacitive properties of materials. The cyclic voltammograms of (S1)_2_Cu and (S2)_2_Cu were obtained in the potential range of 0–0.6 V at various scan rates from 10 mV s^−1^ to 150 mV s^−1^ in 1 M KOH electrolyte (Fig. 7A–C). Interestingly, the CV response of (S1)_2_Cu and (S2)_2_Cu shows both oxidation and reduction peaks at all the scan rates, which indicates the pseudo-capacitive behaviour of these materials. It is found that the current responses from the CV curve increase with an increase in the scan rates for both the materials. Fig. 7C corresponds to the comparative CVs of (S1)_2_Cu and (S2)_2_Cu at a scan rate of 10 mV s^−1^ in the potential range of 0–0.6 V in the electrolyte of 1 M KOH. A pair of oxidation and reduction peaks appeared at 0.46 V and 0.32 V, respectively when the CV of (S1)_2_Cu was measured at a lower scan rate of 10 mV s^−1^. Under similar conditions, the oxidation and reduction peaks of (S2)_2_Cu are displayed at 0.46 V and 0.34 V, respectively. Since the area under the CV curves represents the charge storage capacity, a larger area under the CV curve indicates a higher gravimetric capacitance for (S1)_2_Cu compared to (S2)_2_Cu (Fig. 7C). The diffusion kinetics of ions into (S1)_2_Cu and (S2)_2_Cu was further analyzed using Randles–Sevcik eqn (3).^56^3
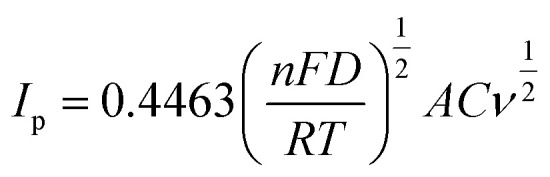
where ‘*I*_p_’ is the peak current, ‘*n*’ stands for the number of electrons transferred, ‘*F*’ is the Faraday constant, ‘*R*’ is the universal gas constant, ‘*T*’ is the absolute temperature, ‘*A*’ corresponds to the area of the working electrode in cm^2^, ‘*C*’ is the concentration of the redox active species and ‘*ν*’ is the scan rate used. The diffusion co-efficient of ions ‘*D*’ was calculated by plotting the peak current against the square root of scan rate and substituting the slope of the linear graph in eqn (3) (Fig. 8). Interestingly, the diffusion coefficients of ions for (S1)_2_Cu are found to be 3.6735 × 10^−4^ and 6.2361 × 10^−4^, respectively, for the cathodic and anodic processes. However, (S2)_2_Cu displays diffusion coefficients of 2.2494 × 10^−4^ and 2.9814 × 10^−4^, respectively, for cathodic and anodic processes. Thus, the higher diffusion coefficient of ions for (S1)_2_Cu can be helpful for its high-rate capacitive performance.

**Fig. 9:**
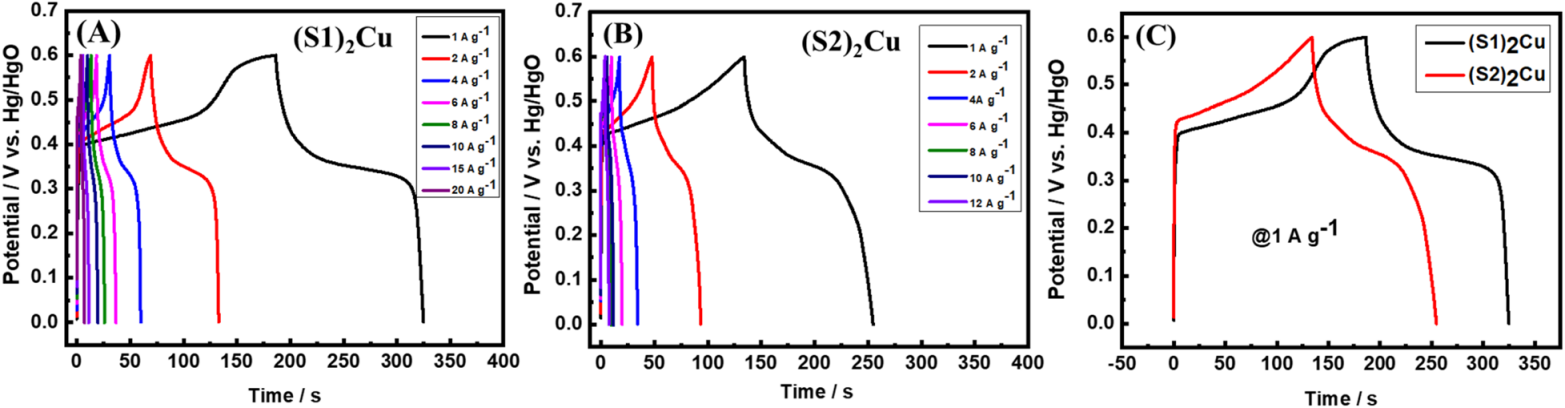
GCD voltage profile of (A) (S1)_2_Cu and (B) (S2)_2_Cu at different specific currents varying from 1 A g^−1^ to 20 A g^−1^ and 1 A g^−1^ to 12 A g^−1^, respectively in the potential range of 0–0.6 V in electrolyte containing 1.0 KOH. (C) Comparative charge–discharge curves of (S1)_2_Cu and (S2)_2_Cu at a specific current of 1 A g^−1^.

**Fig. 10:**
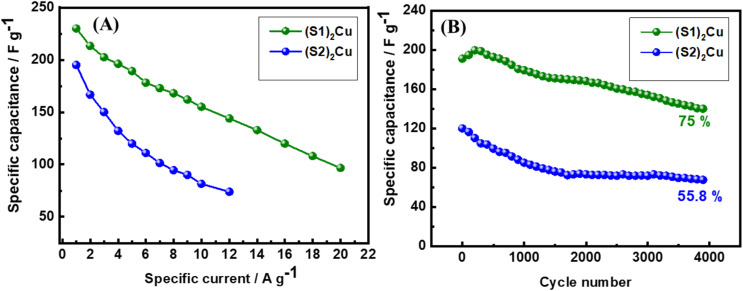
(A) Rate capability test at various specific currents, and (B) long-term cycling performance of (S1)_2_Cu and (S2)_2_Cu at a specific current of 3 A g^−1^ in the potential range of 0–0.6 V in 1 M KOH electrolyte.

In order to calculate the specific capacitances of these materials, the GCD cycling of (S1)_2_Cu and (S2)_2_Cu was conducted at different specific currents varying from 1 A g^−1^ to 10 A g^−1^ in the potential range of 0–0.6 V in 1 M KOH as electrolyte (Fig. 9A and B). The specific capacitances of these materials have been calculated using the following eqn (4);4
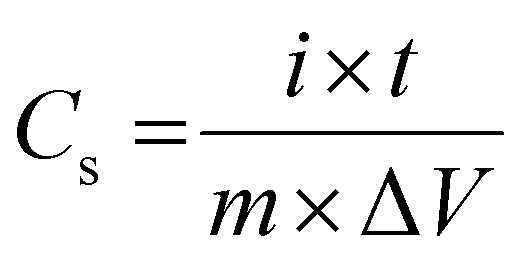
where ‘*C*_s_^’^ is the specific capacitance, ‘*i*’ is the current applied, ‘*t*’ represents time taken for charging or discharging, ‘*m*’ is the active mass of the material and ‘Δ*V*’ is the operational voltage. The specific capacitances of (S1)_2_Cu and (S2)_2_Cu are found to be 230.0 and 195.0 F g^−1^, respectively at a specific current of 1 A g^−1^ in the potential range of 0–0.6 in 1 M KOH electrolyte (Fig. 9C). The specific capacitances of both the materials are found to decrease upon increasing the specific current, which is attributed to the reduced utilization of the active mass at higher currents. The rate performance of (S1)_2_Cu and (S2)_2_Cu was evaluated by performing the galvanostatic cycling at different specific currents. Fig. 10A demonstrates the rate capability of (S1)_2_Cu and (S2)_2_Cu, which clearly displays the decrease in the specific capacitance of both materials with an increase in the specific current. (S1)_2_Cu displayed a higher specific capacitance of 230.0 F g^−1^ at 1 A g^−1^, which decreased to 144.0 F g^−1^ when the specific current increased to 12.0 A g^−1^. Thus, the capacitance retention is about 62.6% of *C*_s_ achieved at 1.0 A g^−1^. Interestingly, (S1)_2_Cu can deliver a *C*_s_ of about 96.6 F g^−1^ even at a higher specific current of 20.0 A g^−1^, where the capacitance retention is about 42%. In the case of (S2)_2_Cu, a *C*_s_ of 195.0 F g^−1^ is achieved at 1.0 A g^−1^, which decreased to 74.0 F g^−1^ upon increasing the specific current to 12 A g^−1^, thus retaining about 37.9% capacitance of that achieved at 1 A g^−1^. Thus, these studies revealed the higher specific capacitance and superior rate performance of (S1)_2_Cu over (S2)_2_Cu. The long-term cyclability of electrode materials is essential for the supercapacitor applications. In this regard, we have examined the long-term cyclability of (S1)_2_Cu and (S2)_2_Cu by performing GCD at a specific current of 3 A g^−1^ for 4000 continuous cycles in the potential range of 0–0.6 V in 1 M KOH electrolyte (Fig. 10B). (S1)_2_Cu exhibited an initial *C*_s_ of 191.0 F g^−1^, which decreased to 144.5 F g^−1^ after 4000 cycles, thus showing a capacitance retention of about 75%. In the case of (S2)_2_Cu, the initial *C*_s_ of 120.0 F g^−1^ decreased to 67.0 F g^−1^ after 4000 cycles, thus retaining about 55.8% capacitance. These results indicated the superior cycling stability of (S1)_2_Cu over (S2)_2_Cu, which is essential for the supercapacitor applications. In order to compare the electrochemical performances of bare Ni foam, we have conducted CV and GCD under similar conditions. The electrochemical performance shows that the specific capacitance of bare Ni foam is (7 F g^−1^) negligible as compared to that of our electrode materials (Fig. S13). Furthermore, we have evaluated the electrochemical performance in high concentration electrolyte for (S1)_2_Cu and (S2)_2_Cu materials in 6.0 M KOH (Fig. S14 and S15). There (S1)_2_Cu and (S2)_2_Cu exhibit higher specific capacitances of 328 F g^−1^ and 268 F g^−1^. However, both electrodes exhibit poor cycling stability in concentrated KOH electrolyte. These results demonstrate that while concentrated alkaline electrolyte enhances charge storage, the cycling stability is found to be severely affected during long-term cycling.

### Performance of the AC||(S1)_2_Cu hybrid supercapacitor

In order to fabricate a hybrid supercapacitor (HSC), AC was used as the −ve electrode and (S1)_2_Cu as the +ve electrode, and its performance was evaluated in 1.0 M KOH electrolyte. Before assembling the asymmetric supercapacitors, the capacitive behaviour of activated carbon (AC) as the negative electrode was evaluated in 1.0 M KOH. As shown in Fig. S16a, the CV curve exhibits a nearly rectangular shape, characteristic of electric double-layer capacitance (EDLC), and with increasing scan rate, the CV shape becomes slightly distorted due to polarization. The specific capacitance of AC, calculated from GCD analysis (Fig. S16b), was found to be 183 F g^−1^ at 1 A g^−1^ within the potential range of −1.0 to 0 V in 1.0 M KOH electrolyte.

For the assembly of a hybrid supercapacitor (HSC), the charge stored on the positive and negative electrodes must be balanced. Therefore, mass balancing between the (S1)_2_Cu and AC electrodes was carried out based on their specific capacitances and operating potential windows. The mass ratio was calculated using the following relation (5):5
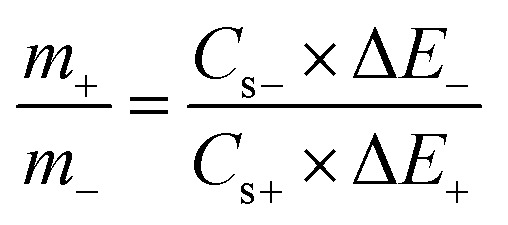
Here, ‘*m*’ denotes the electrode mass, ‘*C*_s_’ represents the specific capacitance, and ‘Δ*E*’ refers to the operating potential window. The subscripts ‘−’ and ‘+’ indicate the negative and positive electrodes, respectively. The optimized mass ratio of the positive to the negative electrode was determined to be 1 : 0.75.

**Fig. 11:**
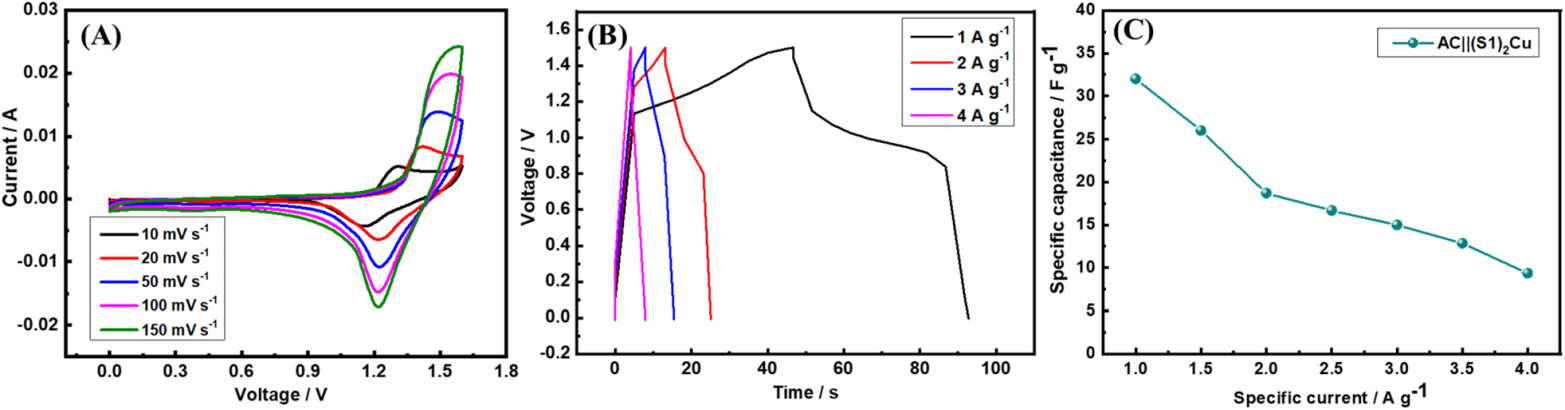
(A) CV of AC||(S1)_2_Cu at different scan rates in the voltage range of 0–1.6 V, (B) GCD of AC||(S1)_2_Cu at different specific currents in the voltage range of 0–1.5 V, and (C) rate performance of AC||(S1)_2_Cu in 1.0 M KOH electrolyte.

The AC||(S1)_2_Cu supercapacitor was assembled, and the CV was performed within the voltage window of 0–1.6 V at scan rates between 10 and 150 mV s^−1^ using 1.0 M KOH electrolyte. The CV profile (Fig. 11A) reveals well-defined redox peaks at 1.25 V (oxidation) and 1.10 V (reduction), demonstrating the contribution of faradaic processes to the charge-storage mechanism. To further analyze its electrochemical behaviour and specific capacitance, GCD experiments were carried out at specific currents ranging from 1 to 4 A g^−1^ in the voltage range of 0–1.5 V (Fig. 11B). The rate performance of the device was assessed by varying the specific currents, as illustrated in Fig. 11C. At 1 A g^−1^, the HSC achieves a specific capacitance of 32 F g^−1^, which gradually decreases to 9.3 F g^−1^ when the specific current increases to 4 A g^−1^.

### Energy density (E.D) and power density (P.D)

The energy density (E.D) and power density (P.D) are essential features for evaluating the performance of supercapacitors. Therefore, these parameters were evaluated for the AC||(S1)_2_Cu HSC using the following eqn (6) & (7).6
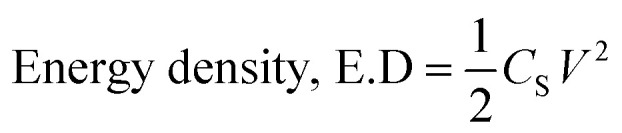
7
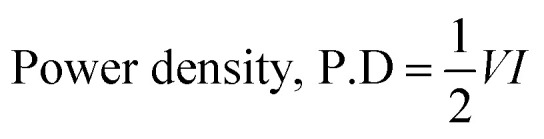
where ‘*C*_S_’ is the specific capacitance, ‘*V*’ stands for operational voltage, and ‘*I*’ corresponds to specific current.

**Fig. 12:**
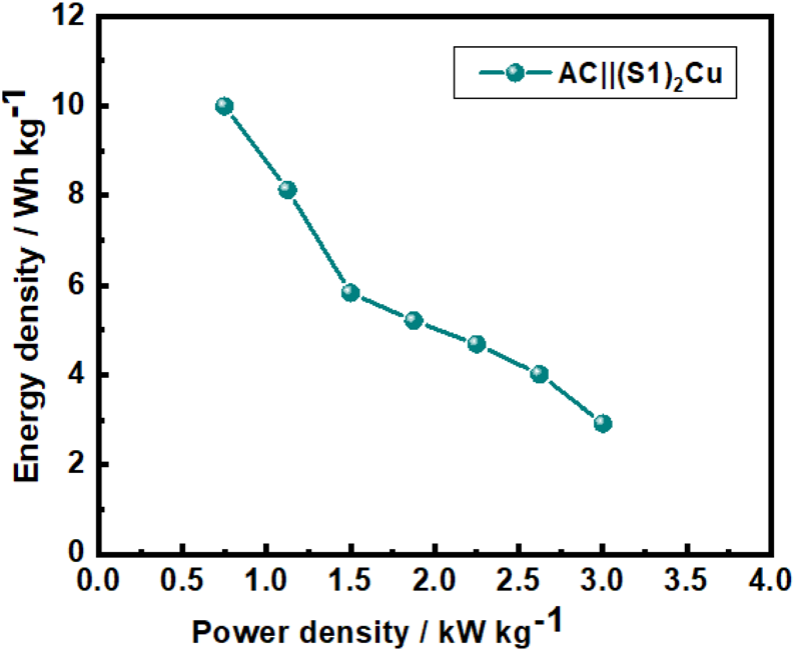
Ragone plot of the AC||(S1)_2_Cu HSC in an aqueous 1.0 M KOH electrolyte.

The Ragone plot of the AC||(S1)_2_Cu HSC derived from the calculated energy and power densities at various current densities is presented in Fig. 12. The device delivers a maximum energy density of 10 Wh kg^−1^ along with a power density of 3 kW kg^−1^. This energy density is comparable to previously reported energy densities of some aqueous hybrid supercapacitors.^73–76^

## Conclusion

Here, we have reported the synthesis of Cu(ii) complexes derived from two phenanthro[9,10-*d*]imidazole-based ligands, S1 and S2, featuring suitable substitutions. These Cu(ii) complexes exhibited self-assembly behaviour, forming well-ordered superstructures with distinct morphological characteristics. The self-assembly process leading to these superstructures was investigated using a combination of microscopic and spectroscopic techniques, along with PXRD analysis. The results revealed that various non-covalent interactions played a crucial role in achieving a lamellar molecular arrangement, followed by a layer closure process, resulting in superstructures with minimal surface energy. Additionally, the potential of these superstructures for VOC adsorption and sensing was evaluated through SKP measurements. Among the findings, the self-assembled structures of these Cu(ii) complexes demonstrated significant selectivity and sensitivity toward acetone detection. The pseudo-capacitive behaviour of (S1)_2_Cu and (S2)_2_Cu based superstructures is investigated in an aqueous electrolyte of 1.0 M KOH and the specific capacitances are found to be 230.0 F g^−1^ and 195.0 F g^−1^, respectively. Additionally, (S1)_2_Cu exhibited superior pseudo-capacitive performance compared to (S2)_2_Cu, including a higher rate performance and better capacitance retention of 75% after 4000 continuous cycles. These results highlight the advantage of (S1)_2_Cu as an electrode material for aqueous supercapacitor applications. Hence, the Cu(ii) complexes (S1)_2_Cu and (S2)_2_Cu are promising smart materials for acetone sensing, offering potential applications in the development of VOC sensor devices and supercapacitor applications.^77,78^

## Materials and methods

### Materials

All the chemicals and solvents used are commercially available and were used as received without further purification. Phenanthrene-9,10-dione, 2-salicylaldehyde, 4-methoxy salicylaldehyde, sodium hydrogen carbonate, and copper chloride were purchased from AVRA. Ammonium acetate was purchased from Sisco Research Laboratories (SRL) Pvt. Ltd, India. Glacial acetic acid, methanol, DMSO, and tetrahydrofuran were purchased from Finar Ltd. *N*-methyl pyrrolidone (NMP) and poly(vinylidene fluoride) (PVDF) were received from Sigma-Aldrich.

### Synthesis of S1, S2, (S1)_2_Cu, and (S2)_2_Cu

The detailed protocol utilized to synthesize S1, S2, (S1)_2_Cu, and (S2)_2_Cu is available in the SI Experimental section (SI Fig. S1–S8).

### Fourier transform infrared spectroscopy (FT-IR)

Fourier transform infrared spectra were recorded using an IR Tracer-100 FT-IR spectrometer (Shimadzu) with a Deuterated Lanthanum α-alanine doped TriGlycine Sulphate (DLaTGS) detector. FT-IR analysis of these newly synthesized (S1)_2_Cu, and (S2)_2_Cu was carried out on the dried mass. The measurements were taken using a resolution of 4 cm^−1^ and an average of 1000 scans. The transmittance minimal values were determined using the Lab solutions IR analysis program (IR Tracer).

### UV-vis spectroscopy

UV-vis absorption spectra of the synthesized (S1)_2_Cu, and (S2)_2_Cu were recorded in a 50% aqueous-ACN medium using a UV-vis spectrophotometer (Shimadzu UV-vis spectrophotometer 1900i).

### Fluorescence spectroscopy

Fluorescence measurements were performed at RT using a fluorescence spectrophotometer (Edinburgh Instruments, FLS 1000). The emission spectra of the synthesized (S1)_2_Cu and (S2)_2_Cu were recorded in an 50% aqueous-ACN medium using appropriate excitation wavelengths.

### High-resolution scanning electron microscopy (HR-SEM)

A 10 μL drop of the respective self-assembled solutions of (S1)_2_Cu and (S2)_2_Cu in a 90% aqueous-ACN medium was placed on a glass coverslip and allowed to dry at RT. HR-SEM analysis was performed using a high-resolution scanning electron microscope (HR-SEM, ThermoScientific Apreo S) operating at 18 kV.

### High-resolution transmission electron microscopy (HR-TEM)

A 10 μL drop of a self-assembled solution of (S1)_2_Cu and (S2)_2_Cu was placed on a 200-mesh copper grid and covered by a carbon stabilized Formvar film. After 1 min, excess fluid was removed from the grid. The samples were analysed using a transmission electron microscope, JEOL-JEM-2100 Plus (high-resolution scintillator) operating at 200 kV.

### X-ray diffraction (XRD) analysis

The PXRD patterns of (S1)_2_Cu and (S2)_2_Cu were recorded using a PANalytical X'Pert Pro Powder X-ray diffractometer. Data collection was carried out at room temperature using Cu Kα radiation (1.5406 Å; 40 kV, 30 mA) as the X-ray source in 2*θ* continuous scan mode (Bragg–Brentano geometry) in the range of 2–50° at a scan rate of 1° min^−1^ and a time per step of 0.5 s.

### Thermogravimetric analysis (TGA)

Thermal analysis of (S1)_2_Cu and (S2)_2_Cu were carried out using a NETZSCH NJA-STA 2500 TGA thermal analyser with a heating rate of 10 °C min^−1^ under an N_2_ atmosphere.

### Preparation of electrodes

The working electrodes for the capacitive performance measurement were prepared by grinding the active material, conducing carbon black and PVDF in a wt% of 75, 15 and 10, respectively. The homogeneous slurry was made by adding a few drops of NMP solvent to the ground powder. Then the slurry was coated onto nickel foam (area 1.0 cm^2^) using a simple brush coating method. Finally, the electrode materials coated on Ni foam were dried in a vacuum oven at a temperature of 100 °C overnight. The active mass of the electrodes is 1.35 and 1.27 mg cm^−2^ for (S1)_2_Cu and (S2)_2_Cu), respectively. Electrochemical capacitive performance of (S1)_2_Cu and (S2)_2_Cu was studied using cyclic voltammetry (CV) and GCD. A three-electrode system was used for the evaluation of capacitive performance using either (S1)_2_Cu or (S2)_2_Cu as the working electrode, Pt foil as the counter electrode and Hg/HgO as the reference electrode. All the electrochemical studies were performed in an aqueous electrolyte of 1.0 M KOH.

## Author contributions

Mallayasamy Siva: analysis and interpretation of data and writing and modification of the manuscript. Aneesh Anand Nechikott: analysis and interpretation of data and writing and modification of the manuscript. Sheethal Sasi: analysis and interpretation of data and writing and modification of the manuscript. Yuvaraj Sivalingam: conceptualisation, design acquisition of data, analysis and interpretation of data, and writing and editing of the manuscript. Prasant Kumar Nayak: conceptualisation, methodology, design acquisition of data, analysis and interpretation of data, writing and editing of the manuscript. Priyadip Das: conceptualisation, methodology, design acquisition of data, analysis and interpretation of data, writing and editing of the manuscript, study supervision, funding acquisition, and project administration.

## Conflicts of interest

There are no conflicts to declare.

## Supplementary Material

NA-007-D5NA00758E-s001

## Data Availability

Characterisation data for the compound along with further supporting data referenced in the manuscript are available in the supplementary information (SI). Supplementary information: detailed synthetic procedures, ^1^H NMR spectra, ^13^C NMR spectra, mass spectra, length distribution, width distribution, crystallinity data, FT-IR analysis, thermogravimetric analysis, 3D raster scan images, cyclic voltammetry and cycling stability studies. See DOI: https://doi.org/10.1039/d5na00758e.

## References

[cit1] Aida T., Meijer E. W., Stupp S. I. (2012). Science.

[cit2] Wurthner F., Moller C. R. S., Fimmel B., Ogi S., Leowanawat P., Schmidt D. (2016). Chem. Rev..

[cit3] Schnizer T., Preuss M. D., Basten J. V., Schoenmakers S. M. C., Spiering A. J. H., Vantomme G., Meijer E. W. (2022). Angew. Chem., Int. Ed..

[cit4] Khasbaatar A., Xu Z., Lee J. J., Alvarado G. C., Hwang C., Onusaitis B. N., Diao Y. (2023). Chem. Rev..

[cit5] Kato T., Yoshio M., Ichikama T., Soberats B., Ohno H., Funahashi M. (2017). Nat. Rev. Mater..

[cit6] Zhang Z., Mu B., Miao X., Wang L., Lu H., Ma Y., Tian W. (2024). Chem.

[cit7] Kobaisi M. A., Bhosale S. V., Latham K., Raynor A. M., Bhosale S. V. (2016). Chem. Rev..

[cit8] Whitesides G. W., Grzybowski B. (2002). Science.

[cit9] Zang L., Che Y., Moore J. S. (2008). Acc. Chem. Res..

[cit10] Haedler A. T., Kreger K., Issac A., Wittmann B., Kivala M., Hammer N., Kohler J., Schmidt H. W., Hildner R. (2015). Nature.

[cit11] Miyajima D., Araoka F., Takezoe H., Kim J., Kato K., Takata M., Aida T. (2012). Science.

[cit12] Zhao Y. S., Fu H., Peng A., Ma Y., Liao Q., Yao J. (2010). Acc. Chem. Res..

[cit13] Zheng H., Li Y., Liu H., Yin X., Li Y. (2011). Chem. Soc. Rev..

[cit14] Hoeben F. J. M., Jonkheijm P., Meijer E. W., Schenning A. P. H. J. (2005). Chem. Rev..

[cit15] Maity A., Ali F., Agarwalla H., Anothumakkool B., Das A. (2015). Chem. Commun..

[cit16] Mahapatra T. S., Singh H., Maity A., Dey A., Pramanik S. K., Suresh E., Das A. (2018). J. Mater. Chem. C.

[cit17] Lakshmanan A., Zhang S., Hauser C. A. E. (2012). Trends Biotechnol..

[cit18] Li G., Wu Y., Gao J., Wang C., Li J., Zhang H., Zhao Y., Zhao Y., Zhang Q. (2012). J. Am. Chem. Soc..

[cit19] Zou Q., Liu K., Abbas M., Yan X. (2016). Adv. Mater..

[cit20] Agarwalla H., Pal S., Paul A., Jun J. W., Bae J., Ahn K. H., Srivastava K. D. N., Das A. (2016). J. Mater. Chem. B.

[cit21] Das P., Bhattacharya S., Mishra S., Das A. (2011). Chem. Commun..

[cit22] Rana P., Marappan G., Sivagnanam S., Surya V. J., Sivalingam Y., Das P. (2022). Mater. Chem. Front..

[cit23] Dimitrakopoulos C. D., Malenfant P. R. L. (2002). Adv. Mater..

[cit24] Braun D. (2002). Mater. Today.

[cit25] Friend R. H., Gymer R. W., Holmes A. B., Burroughes J. H., Marks R. N., Taliani C., Bradley D. D. C., Dos Santos D. A., Bredas J. L., Logdlund M., Salaneck W. R. (1999). Nature.

[cit26] Brabec C. J. , Dyakonov V., Parisi J. and Sariciftci N. S., Springer Series in Materials Science, Springer Berlin Heidelberg, Berlin, Heidelberg, 2003, vol. 60, 10.1007/978-3-662-05187-0

[cit27] Das P., Pan I., Cohen E., Reches M. (2018). J. Mater. Chem. B.

[cit28] Dong L., Gao Z., Lin N. (2016). Prog. Surf. Sci..

[cit29] Panda D., Tseng T. Y. (2013). J. Mater. Sci..

[cit30] Yan X., Zhu P., Li J. (2010). Chem. Soc. Rev..

[cit31] Li Y., Liu T., Liu H., Tian M. Z., Li Y. (2014). Acc. Chem. Res..

[cit32] Wu W., Liu Y., Zhu D. (2010). Chem. Soc. Rev..

[cit33] Li S., Zhang W., Xing R., Yuan C., Xue H., Yan X. (2021). Adv. Mater..

[cit34] Liu Y., Naumenko E., Akhatova F., Zou Q., Fakhrullin R., Yan X. (2021). Chem. Eng. J..

[cit35] Siva M., Sasi S., Rana P., Bera R. K., Sivalingam Y., Das P. (2024). New J. Chem..

[cit36] Liu Y., Yao D., Zhang H. (2018). ACS Appl. Mater. Interfaces.

[cit37] Bello F. D., Pellei M., Bagnarelli L., Santini C., Giorgioni G., Piergentlli A., Quaglia W., Battoccgio C., Lucci G., Schiesara I., Meneghini C., Venditti I., Ramanan N., Franco M. D., Sgarbossa P., Marzano C., Gandin V. (2022). Inorg. Chem..

[cit38] Muslim M., Sultan, Kamran L. A., Basree, Pradhan A. K., Alam M. J., Afzal S. M., Ahmad M., Afzal M. (2025). RSC Adv..

[cit39] Hu X. P., Deng W., Lu H. L., Tong J., Yu S. Y. (2021). Inorg. Chem. Commun..

[cit40] Tandon S. S., Bunge S. D., Patel N., Wang E. C., Thompson L. K. (2020). Molecules.

[cit41] Tong J., Lu H. L., Sun W. Q., Yu S. Y. (2020). CrystEngComm.

[cit42] Choi W., Bera R. K., Han S. W., Park H., Go T. W., Choi M., Ryoo R., Park J. Y. (2022). Carbon.

[cit43] Park H., Bera R. K., Yoon H., Kim K. (2025). ACS Appl. Energy Mater..

[cit44] Biswas A. K., Barik S., Sen A., Das A., Ganguly B. (2014). J. Phys. Chem. C.

[cit45] Dey A., Ramlal V. R., Sankar S. S., Kundu S., Mandal A. K., Das A. (2021). Chem. Sci..

[cit46] Dey A., Ramlal V. R., Sankar S. S., Mahapatra T. S., Suresh E., Kundu S., Mandal A. K., Das A. (2020). ACS Appl. Mater. Interfaces.

[cit47] Sarangapan S., Tilak B. V., Chen C. P. (1996). J. Electrochem. Soc..

[cit48] Shao Y., El-Kady M. F., Sun J., Li Y., Zhang Q., Zhu M., Wang H., Dunn B., Kaner R. B. (2018). Chem. Rev..

[cit49] Warren R., Sammoura F., Tounsi F., Sanghadasa M., Lin L. (2015). J. Mater. Chem. A.

[cit50] Nayak P. K. (2012). J. Mater. Sci. Eng. B.

[cit51] Meng G., Yang Q., Wu X., Wan P., Li Y., Lei X., Sun X., Liu J. (2016). Nano Energy.

[cit52] Wang G., Huang J., Chen S., Gao Y., Cao D. (2011). J. Power Sources.

[cit53] Yang J., Lan T., Liu J., Song Y., Wei M. (2013). Electrochim. Acta.

[cit54] Zhang H. X., Feng J., Zhang M. L. (2008). Mater. Res. Bull..

[cit55] Li Y., Chang S., Liu X., Huang J., Yin J., Wang G., Cao D. (2012). Electrochim. Acta.

[cit56] Moosavifard S. E., El-Kady M. F., Rahmanifar M. S., Kaner R. B., Mousavi M. F. (2015). ACS Appl. Mater. Interfaces.

[cit57] Hsu Y. K., Chen Y. C., Lin Y. G. (2012). J. Electroanal. Chem..

[cit58] Pramanik A., Maiti S., Mahanty S. (2015). Dalton Trans..

[cit59] Shinde S. K., Dubal D. P., Ghodake G. S., Kim D. Y., Fulari V. J. (2014). J. Electroanal. Chem..

[cit60] Jeyaraj P., Siva M., Selvaraj E., Das P., Baskar B. (2024). Asian J. Org. Chem..

[cit61] Teng T., Xiong J., Cheng G., Zhou C., Lv X., Li K. (2021). Molecules.

[cit62] Das P., Ghosh A., Kesharwani M. K., Ramu V., Ganguly B., Das A. (2011). Eur. J. Inorg. Chem..

[cit63] Lee H. N., Sway K. M. K., Kim S. K., Kwon J. Y., Kim Y., Kim S. J., Yoon Y. J., Yoon J. (2007). Org. Lett..

[cit64] Rana P., Jennifer A. G., Siva M., Varathan E., Das P. (2022). New J. Chem..

[cit65] Pellei M., Del Bello F., Porchia M., Santini C. (2021). Zinc Coordination Complexes as Anticancer Agents. Coord. Chem. Rev..

[cit66] Maity A., Dey A., Gangopadhya M., Das A. (2018). Nanoscale.

[cit67] Sasi S., Palanisamy P., Reji R. P., Nutalapati V., Jayaraman S. V., Kawazoe Y., Sivalingam Y. (2024). ACS Appl. Mater. Interfaces.

[cit68] Matada M. S. S., Kuppuswamy G. P., Martinez M. S., Ghuge R. S., Jayaraman S. V., Sivalingam Y. (2024). ACS Appl. Electron. Mater..

[cit69] Sarngan P. P., Sasi S., Mukherjee P., Mitra K., Sivalingam Y., Swami A., Ghorai U. K., Sarkar D. (2024). Nanoscale.

[cit70] Selvaraju N., Sasi S., Sivalingam Y., Venugopal G. (2024). Diamond Relat. Mater..

[cit71] Sasi S., Marappan G., Sivalingam Y., Chandran M., Magna G., Jayaraman S. V., Paolesse R., Natale C. D. (2024). Surf. Interfaces..

[cit72] Matada M. S. S., Kuppuswamy G. P., Sasi S., Jayaraman S. V., Nutalapat V., Kumar S. S., Sivalingam Y. (2024). ACS Appl. Mater. Interfaces.

[cit73] Ghuge R. H., Reji R. P., Mahata M. S. S., Jayaraman S. V., Magna G., Paolesse R., Sivalingam Y., Natale C. D. (2024). ACS Appl. Nano Mater..

[cit74] Palanisamy P., Anandan M., Sasi S., Bora A., Reji R. P., Balaji S. K. C., Kawazoe Y., Raman G., Jayaraman S. V., Sivalingam Y., Nutalapatti V. (2025). Sustainable Mater. Technol..

[cit75] Salleh N. A., Kheawhom S., Mohamad A. A. (2020). Arab. J. Chem..

[cit76] Mathaiyan R., Nechikott A. A., Sajith B. M. K., Nayak P. K., Kancharla S. (2024). J. Mater. Chem. A.

[cit77] Kuang M., Li T. T., Chen H., Zhang S. M., Zhang L. L., Zhang Y. X. (2015). Nanotechnology.

[cit78] Cai D., Wang D., Liu B., Wang Y., Liu Y., Wang L., Li H., Huang H., Li Q., Wang T. (2013). ACS Appl. Mater. Interfaces.

