# Supplementary material for: Self-assembled π-conjugated Cu(ii)–phenanthro[9,10-d]imidazole superstructures for VOC sensing and enhanced supercapacitor performance
Source: Nanoscale Adv. 2025 Sep 23;7(23):7606–19. doi: 10.1039/d5na00758e (PMC12525573; doi:10.1039/d5na00758e)
Supplement: NA-007-D5NA00758E-s001 [file NA-007-D5NA00758E-s001.pdf]

Supplementary Information (SI) for Nanoscale Advances.
This journal is © The Royal Society of Chemistry 2025

Supplementary Information

Self-Assembled π-Conjugated Cu(II)–Phenanthro[9,10-d]imidazole Superstructures for VOC Sensing and Enhanced Supercapacitor Performance

Mallayasamy Siva[a], Aneesh Anand Nechikott[a], Sheethal Sasi[b], Yuvaraj Sivalingam[c]*, Prasant Kumar Nayak[a]* and Priyadip Das[a]*

[a]Department of Chemistry, SRM Institute of Science and Technology, SRM Nagar, Potheri, Kattankulathur, Tamil Nadu-603203

[b]Department of Physics and Nanotechnology, Faculty of Engineering and Technology, SRM Institute of Science and Technology, Kattankulathur 603203, Tamil Nadu, India.

[c]Centre for Advanced Translational Research, KPR College of Arts Science and Research, Avinashi Road, Arasur, Coimbatore, 641407, Tamil Nadu, India.

* Corresponding author

*Email:priyadipcsmcri@gmail.com, priyadip@srmist.edu.in, prasantnayak15@gmail.com, prasantn1@srmist.edu.in, yuvaraj.sst@gmail.com and yuvaraj.s@kprcas.ac.in

Experimental Section:

Synthesis of S1: A mixture of 2-hydroxy benzaldehyde (293 mg, 2.40 mmol), Phenanthrene-9,10-dione (500 mg, 2.40 mmol), and ammonium acetate (2.4 gm, 31.13 mmol) in glacial acetic acid (15 mL) was heated at 100°C for 12 h with continuous stirring. The hot reaction mixture was cooled to room temperature, whereupon a white solid was precipitated. This white solid was collected by filtration and washed with dilute aqueous $NaHCO_3$ solution followed by distilled water. This white residue was dried and purified by column chromatography on silica gel eluting with Hexane/$CHCl_3$ (95:5, v/v). Yield = 561 mg, (1.8 mmol,75.4%) (Scheme S1). (^{1}H NMR, $CDCl_3$, 500 MHz, δppm): 11.47 (s, 1H), 8.65 (t, *J* = 8.7Hz, 2H), 8.41 (d, *J* = 7.9Hz, 1H), 8.24 (d, *J*= 7.1Hz, 1H), 8.10 (q, *J*=1.35Hz, *J*=6.45Hz, 1H), 7.72-7.63 (m, 4H), 7.45-7.42 (m, 1H), 7.16 (d, *J*=8.25Hz, 1H), 7.04 (t, *J*=7.5Hz, 1H). ^{13}C NMR (100 MHz, $CDCl_3$): δ (ppm): 161.75, 158.05, 143.30, 133.36, 132.98, 129.33, 128.97, 127.56, 127.43, 126.69, 126.63, 126.45, 123.74, 123.46, 122.79, 120.82, 120.60, 119.64, 117.41, 111.07. ESI-MS (m/z): [M-H]$^+$ =309.3407 (calculated); 309.6000 (observed).

Synthesis of S2: A mixture of 2-hydroxy4-methoxy benzaldehyde (365 mg, 2.40 mmol), Phenanthrene-9,10-dione (500 mg, 2.40 mmol), and ammonium acetate (2.4 gm, 31.13 mmol) in glacial acetic acid (15 mL) was heated at 100°C for 12 h with continuous stirring. The hot

reaction mixture was cooled to room temperature, where upon a white solid was precipitated. This white solid was collected by filtration and washed with dilute aqueous $NaHCO_3$ solution followed by distilled water. This white residue was dried and purified by column chromatography on silica gel eluting with Hexane/$CHCl_3$ (95:5, v/v). Yield = 450 mg, (1.32 mmol, 55.07%) (Scheme S1) (^{1}H NMR, $CDCl_3$, 500 MHz, δppm): 11.67 (s, 1H), 8.72(t, *J*= 8.15Hz, 2H), 8.48 (d, *J*= 7.7Hz, 1H), 8.29 (d, *J*= 7.7Hz, 1H), 8.03 (d, *J*= 8.7Hz, 1H), 7.75-7.67 (m, 4H), 6.67-6.62 (m, 2H), 3.89 (s, 3H). ^{13}C NMR (100 MHz, d_6-DMSO): δ (ppm): 158.31, 158.26, 149.75, 137.50, 132.11, 130.47, 128.14, 128.07, 127.69, 127.58, 125.88, 125.69, 124.43, 124.36, 124.34, 122.65, 122.42, 117.49, 116.88, 113.52, 31.14. $[M+2H]^+$ =342.3905 (calculated); 342.1000 (observed).

Synthesis of (S1)$_2$Cu: A mixture of ligand **S1** (200 mg, 0.65 mmol) and cupric chloride ($CuCl_2$) (43.6 mg, 0.32 mmol) in dry methanol (30 mL) was stirred at room temperature for 24 h. On completion of reaction, the precipitate was separated as white-coloured solid which was filtered, dried. Yield: 187 mg (0.27 mmol, 85.4%). ESI-MS (m/z): $[M+2H]^+$ = 684.25 (calculated); 683.95 (observed).

Synthesis of (S2)$_2$Cu: A mixture of **S2** (200 mg) and Cupric chloride (0.28 mmol) in dry methanol (30 mL) was stirred at room temperature for 24 hours. On completion of reaction, the precipitate was separated as white-coloured solid which was filtered, dried. Yield: 172 mg (0.23 mmol, 79.7%). ESI-MS (m/z): $[M]^+$ = 744.2953 (calculated); 744.3500 (observed).

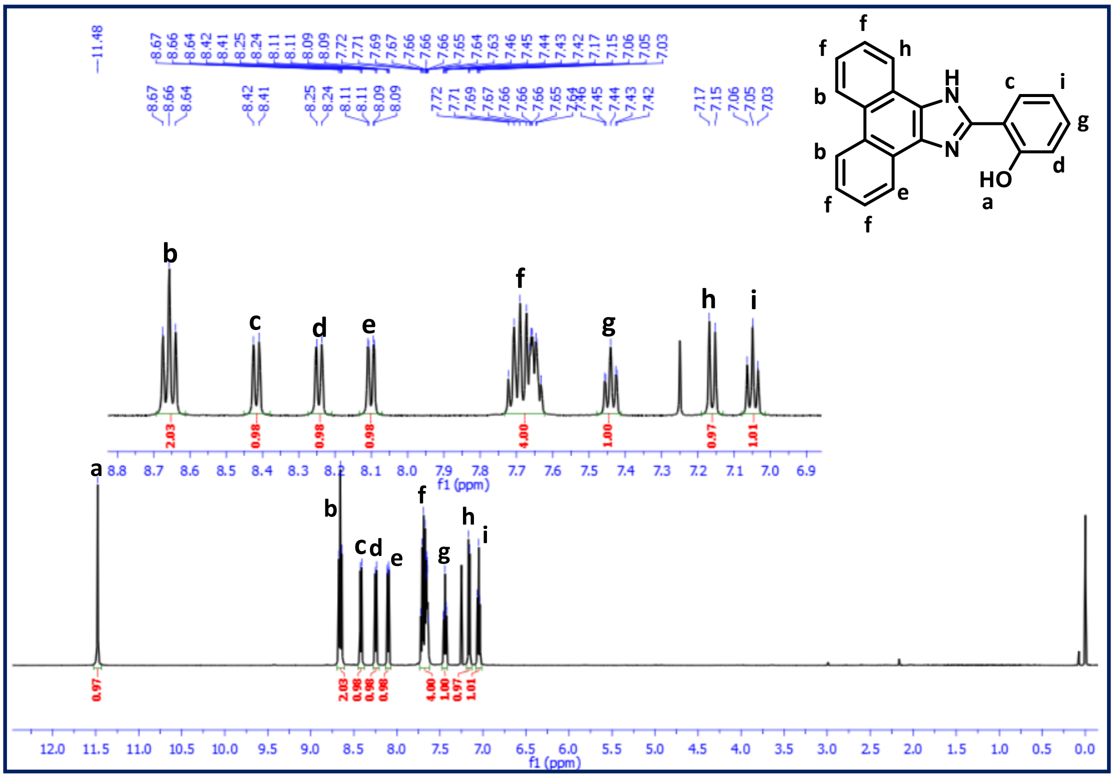

Figure S1. ^{1}H-NMR spectra of **S1** in $CDCl_3$.

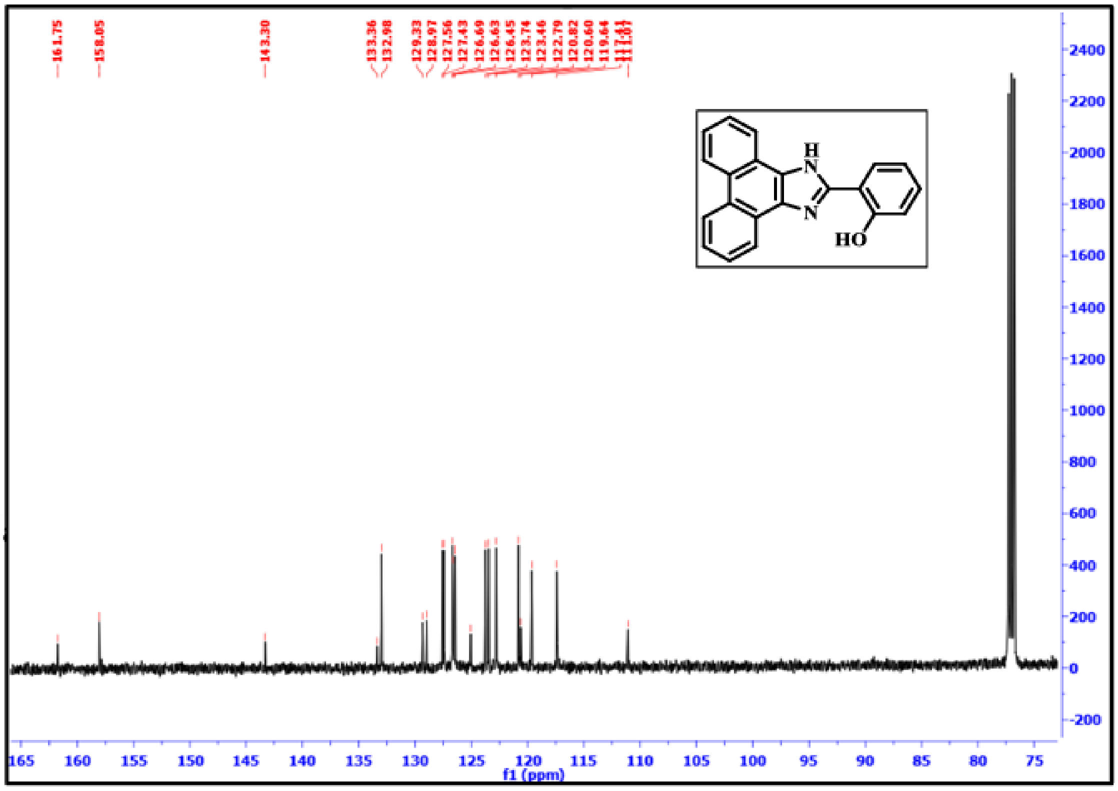

Figure S2. ^{13}C-NMR spectra of **S1** in $CDCl_3$.

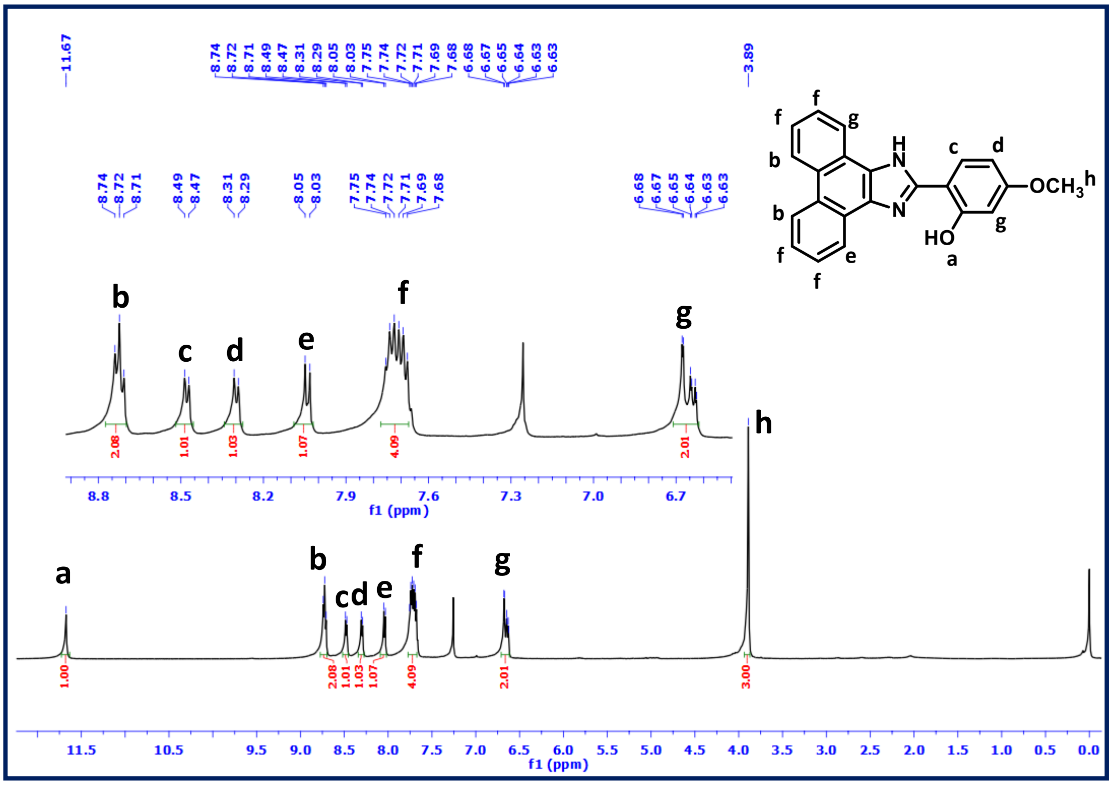

Figure S3. ^{1}H-NMR spectra of **S2** in $CDCl_3$.

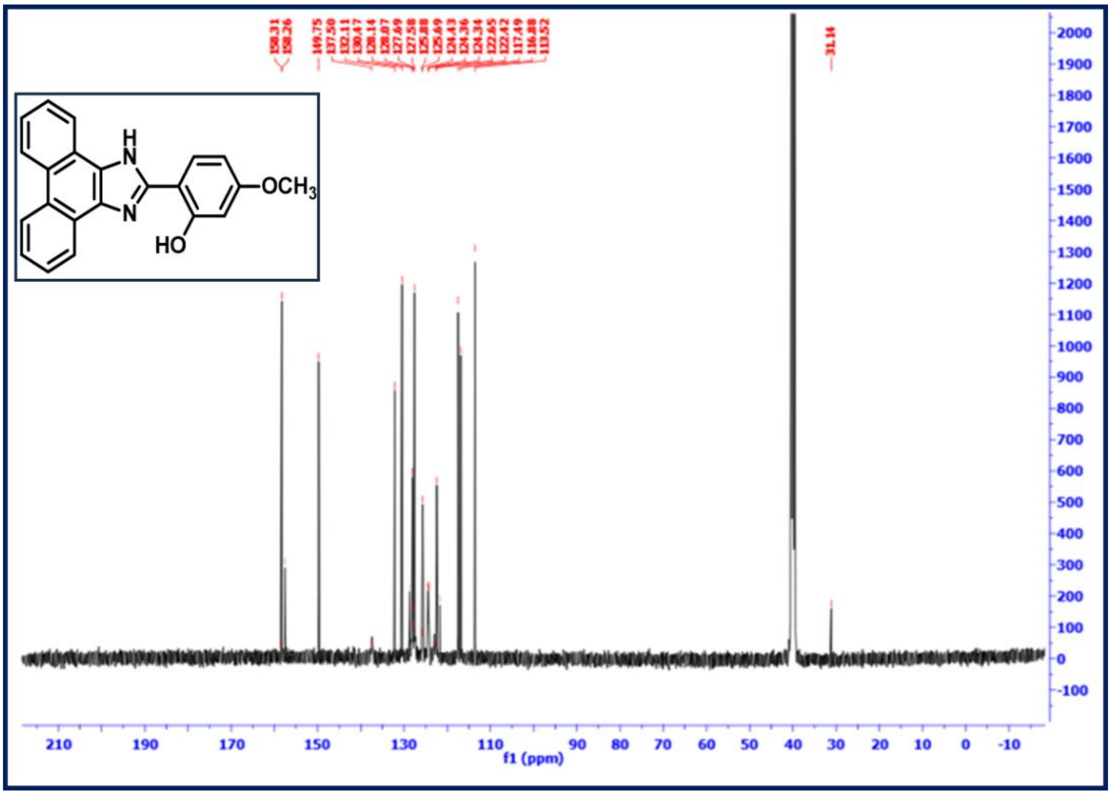

Figure S4. ^{13}C-NMR spectra of **S2** in d_6-DMSO.

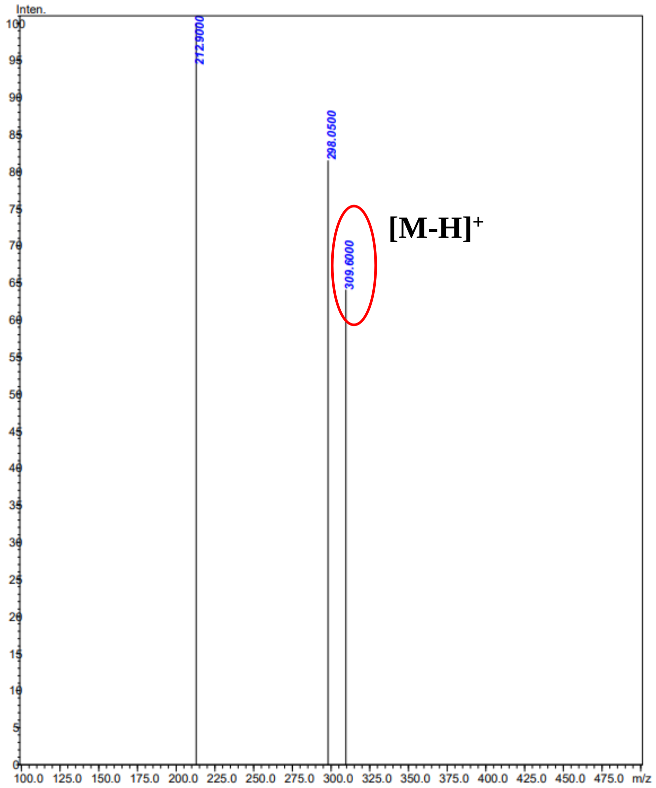

Figure S5. ESI Mass spectra of **S1**.

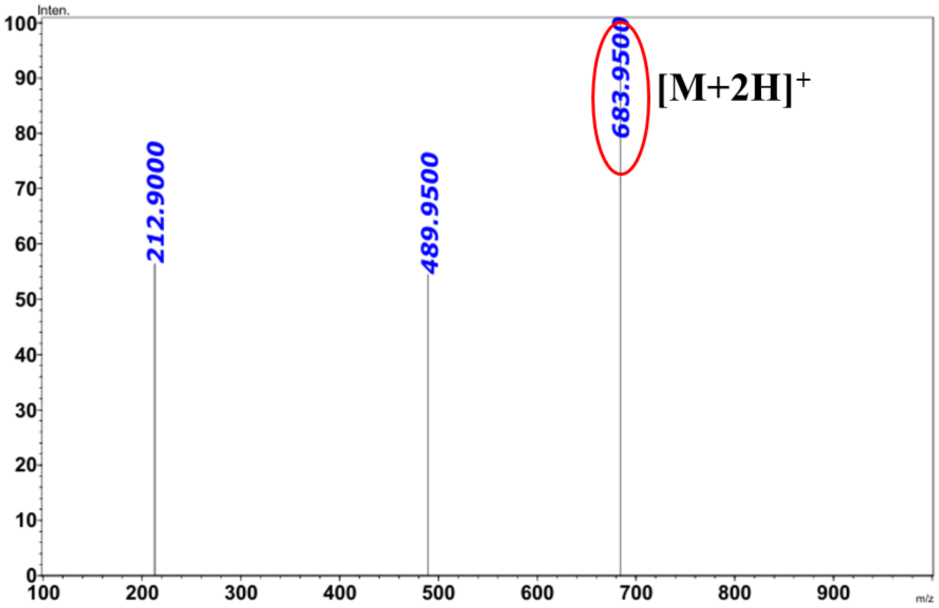

Figure S6. ESI Mass spectra of **$(S1)_2Cu$**.

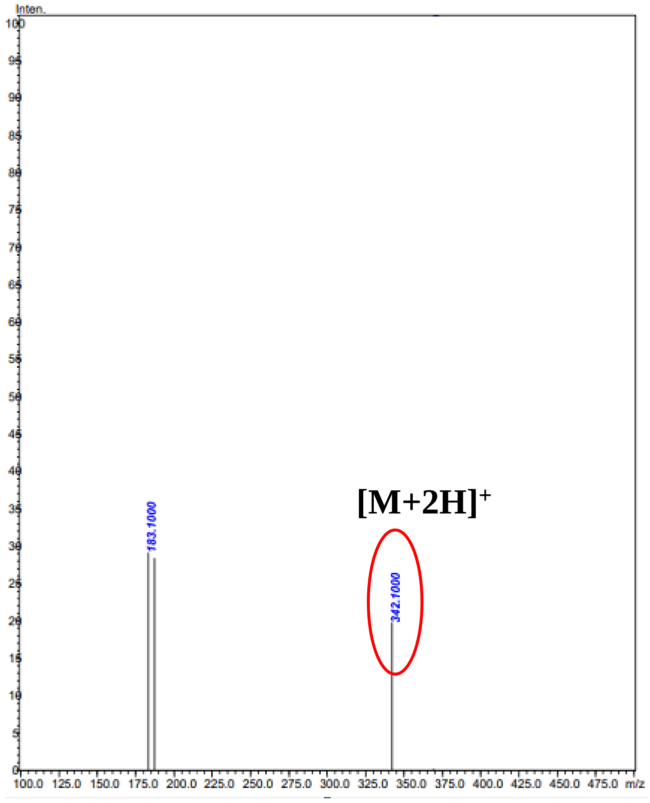

Figure S7. ESI Mass spectra of **S2**.

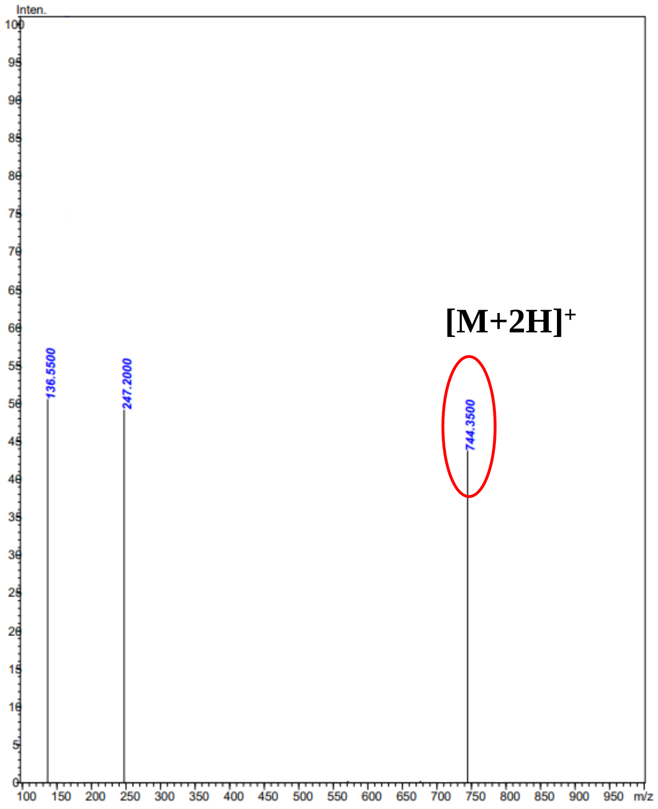

Figure S8. ESI Mass spectra of **(S2)$_2$Cu**.

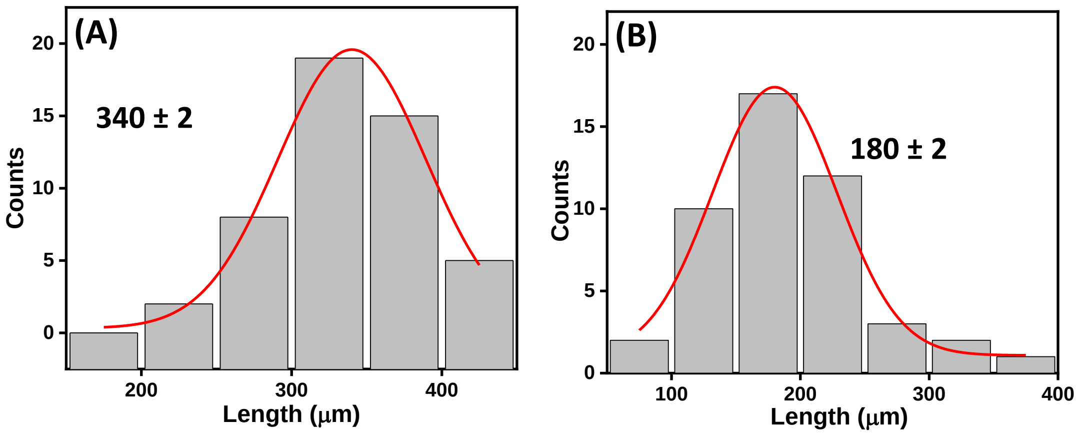

Figure S9. Length distribution of (A) **$(S1)_2Cu$**, and (B) **$(S2)_2Cu$** obtained from HR-SEM images.

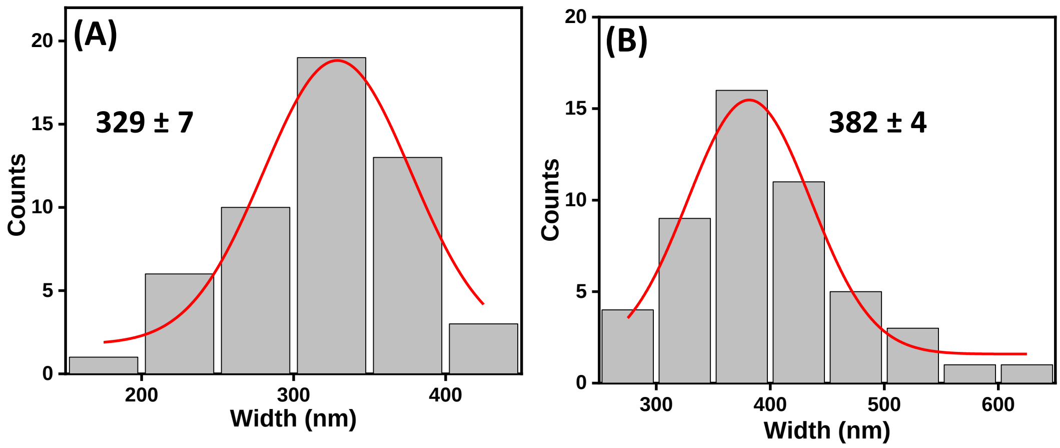

Figure S10. Width distribution of (A) **$(S1)_2Cu$**, and (B) **$(S2)_2Cu$** obtained from HR-SEM images.

Table S1: Crystallinity (%) data of **$(S1)_2Cu$**, and **$(S2)_2Cu$.**

**Metal complexes**	**Crystallinity (%)**
**$(S1)_2Cu$**	56.55
**$(S2)_2Cu$**	78.86

Table S2: Concentration dependent changes in the FT-IR spectral value for ν (C=C str, aromatic), ν (C=N str, aromatic), ν (C-H str, aromatic) and ν (N-H str, imidazole) obtained from dried mass of **$(S1)_2Cu$**, and **$(S2)_2Cu$**.

	**$(S1)_2Cu$**		**$(S2)_2Cu$**	
**Concentration (mg mL^{-1})**	0.5	2.5	0.5	2.5
ν(C=C, aromatic) cm^{-1}	1569	1603	1590	1570
ν(C=N, aromatic) cm^{-1}	1585	1625	1635	1625
ν(C-H, aromatic) cm^{-1}	2902	2920	2850	2830
ν(N-H, imidazole) cm^{-1}	2970	2998	2921	2902

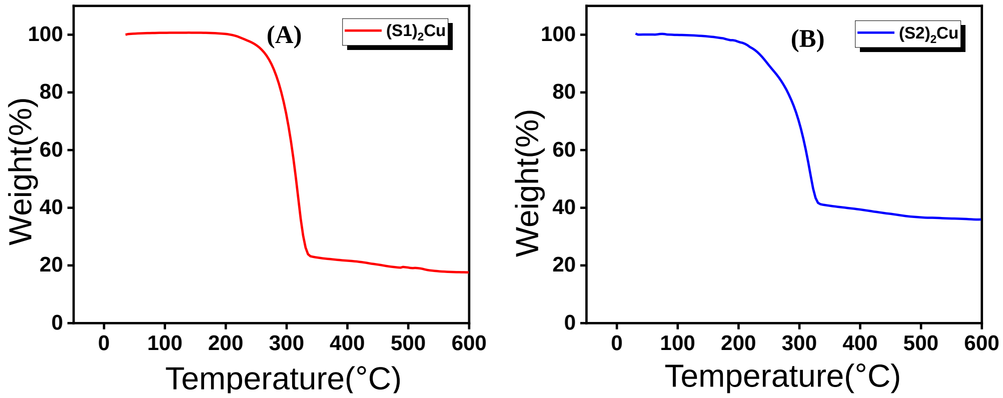

Figure S11. TGA analysis of (A) **(S1)₂Cu**, and (B) **(S2)₂Cu.**

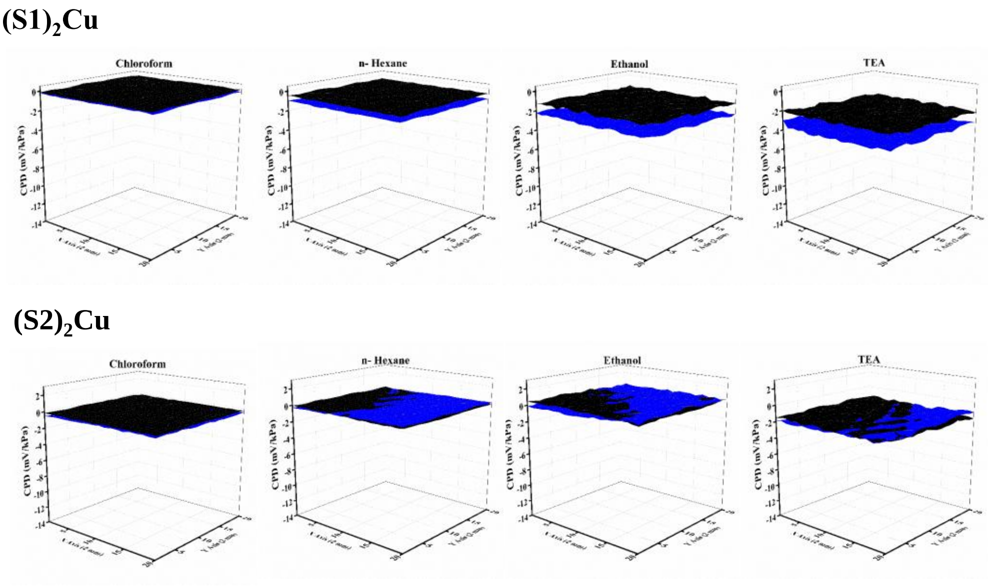

Figure S12. The 3D raster scan CPD measurements of the samples under dark (black) and UV light (blue) illumination for various VOC exposures.

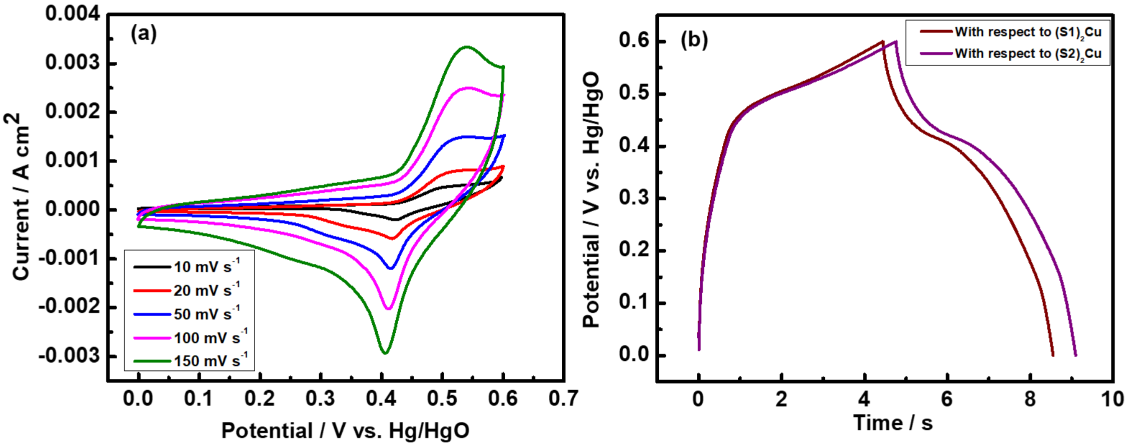

Figure S13 (a) Cyclic voltammetry (CV) curves of bare Ni foam recorded at various scan rates in the potential range of 0 – 0.6 V in 1.0 M KOH electrolyte, and (b) galvanostatic charge–discharge (GCD) profiles obtained at a specific current of 1 A g^{-1} (calculated with respect to the active masses of **(S1)₂Cu** and **(S1)₂Cu**) within the same potential window.

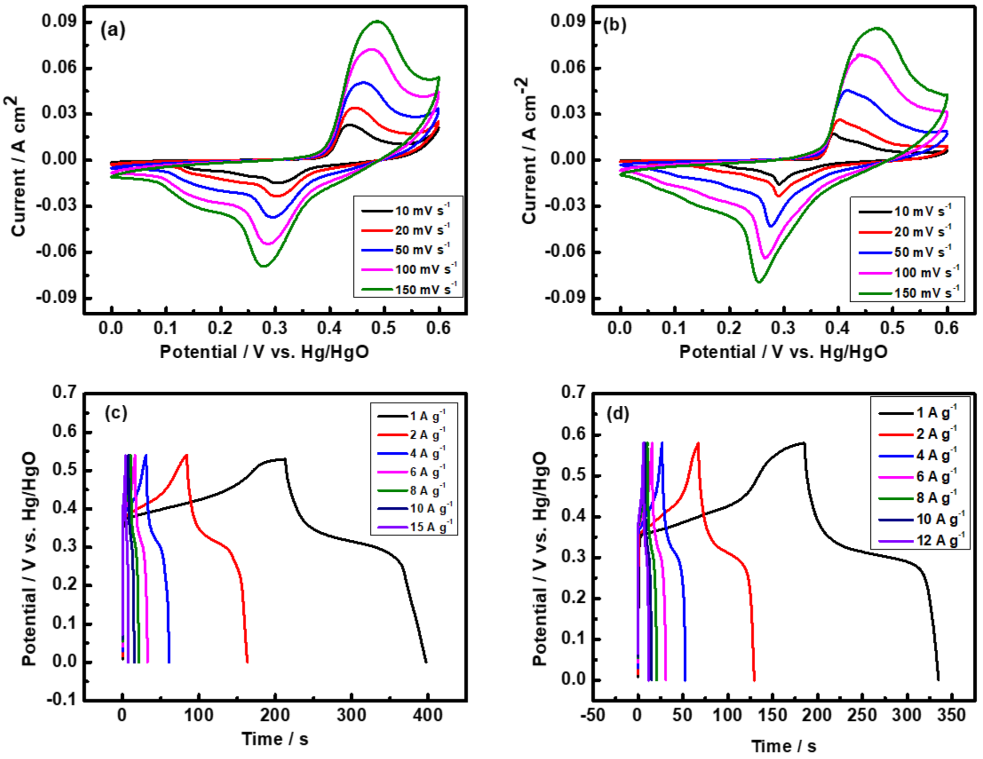

Figure S14 CV of (a) **(S1)₂Cu** and (b) **(S2)₂Cu** at different scan rates in the potential range of 0 – 0.6 V in 6.0 M KOH. GCD at different specific currents in (c) **(S1)₂Cu** in the potential range of 0 – 0.54 V and (d) **(S2)₂Cu** in the potential range of 0 – 0.58V in 6.0 M KOH.

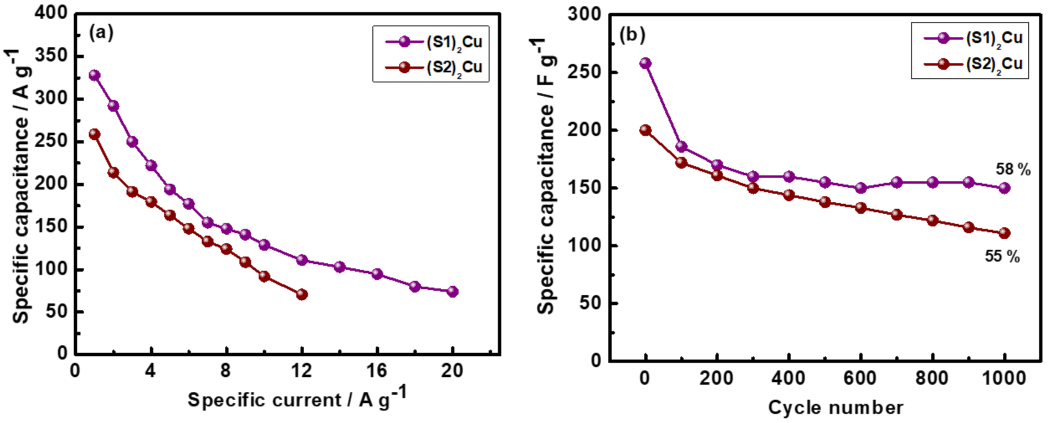

Figure S15 (a) rate capability and (b) cycling stability of **(S1)$_2$Cu** and **(S2)$_2$Cu** at a specific current of 3 A g^{-1} in 1.0 M KOH.

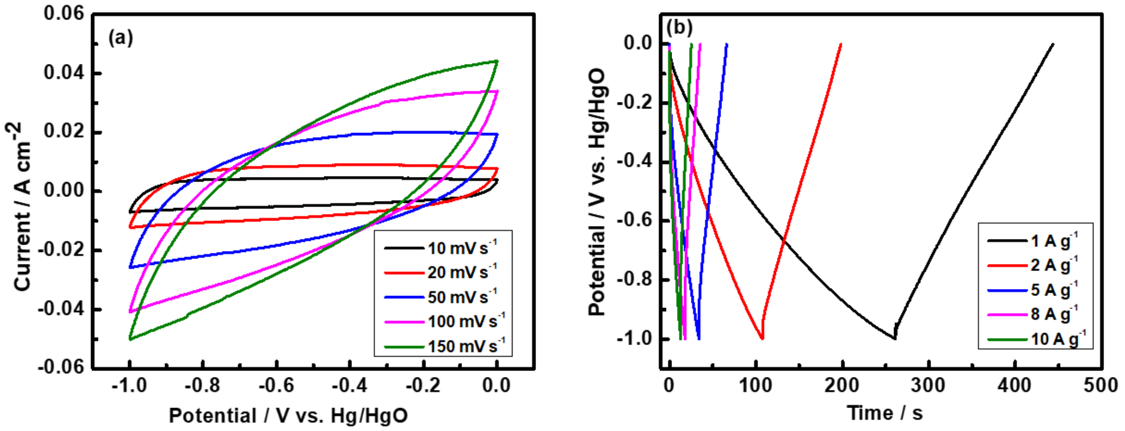

Figure S16 (a) CV of AC at different scan rates and (b) GCD of AC at different specific currents in the potential range of -1.0 to 0 V in 1.0 M KOH electrolyte.
